# Bacteria Contribute to Sediment Nutrient Release and Reflect Progressed Eutrophication-Driven Hypoxia in an Organic-Rich Continental Sea

**DOI:** 10.1371/journal.pone.0067061

**Published:** 2013-06-25

**Authors:** Hanna Sinkko, Kaarina Lukkari, Leila M. Sihvonen, Kaarina Sivonen, Mirja Leivuori, Matias Rantanen, Lars Paulin, Christina Lyra

**Affiliations:** 1 Department of Food and Environmental Sciences, University of Helsinki, Helsinki, Finland; 2 Marine Research Centre, Finnish Environment Institute, Helsinki, Finland; 3 National Institute for Health and Welfare, Helsinki, Finland; 4 Reference Laboratory, Finnish Environment Institute, Helsinki, Finland; 5 Institute of Biotechnology, University of Helsinki, Helsinki, Finland; Dowling College, United States of America

## Abstract

In the sedimental organic matter of eutrophic continental seas, such as the largest dead zone in the world, the Baltic Sea, bacteria may directly participate in nutrient release by mineralizing organic matter or indirectly by altering the sediment’s ability to retain nutrients. Here, we present a case study of a hypoxic sea, which receives riverine nutrient loading and in which microbe-mediated vicious cycles of nutrients prevail. We showed that bacterial communities changed along the horizontal loading and vertical mineralization gradients in the Gulf of Finland of the Baltic Sea, using multivariate statistics of terminal restriction fragments and sediment chemical, spatial and other properties of the sampling sites. The change was mainly explained by concentrations of organic carbon, nitrogen and phosphorus, which showed strong positive correlation with *Flavobacteria*, *Sphingobacteria*, *Alphaproteobacteria* and *Gammaproteobacteria*. These bacteria predominated in the most organic-rich coastal surface sediments overlain by oxic bottom water, whereas sulphate-reducing bacteria, particularly the genus *Desulfobacula*, prevailed in the reduced organic-rich surface sediments in the open sea. They correlated positively with organic nitrogen and phosphorus, as well as manganese oxides. These relationships suggest that the bacterial groups participated in the aerobic and anaerobic degradation of organic matter and contributed to nutrient cycling. The high abundance of sulphate reducers in the surficial sediment layers reflects the persistence of eutrophication-induced hypoxia causing ecosystem-level changes in the Baltic Sea. The sulphate reducers began to decrease below depths of 20 cm, where members of the family *Anaerolineaceae* (phylum *Chloroflexi*) increased, possibly taking part in terminal mineralization processes. Our study provides valuable information on how organic loading affects sediment bacterial community compositions, which consequently may maintain active nutrient recycling. This information is needed to improve our understanding on nutrient cycling in shallow seas where the dead zones are continuously spreading worldwide.

## Introduction

In continental seas receiving nitrogen, phosphorus and organic inputs, such as the Baltic Sea, one of the world’s eutrophic seas with the largest hypoxic dead zone, accelerated primary production results in excess organic matter in sediments [1], [2]. The consequent hypoxia destroys the benthos and interrupts the upward flow of energy in the food chain, which, instead, is directed downwards, feeding microbes in sediment [2]. Simultaneously, the consequent hypoxia-induced release of nutrients such as phosphorus and nitrogen from deposited organic matter can be substantial and sustain the vicious consequences of external loading [3], [4]. The release of phosphorus from iron oxyhydroxides in the reduced sediment is widely investigated since the studies of Mortimer [5], [6], also in the Baltic (e.g. [7]–[10]). Hypoxia can also enhance release of phosphorus from organic compounds in the sediment [11], [12] but this phenomenon has been far less investigated [13], [14].

Phosphate [13], [15], as well as ammonium, is released from organic compounds by bacterial degradation. Generally, sediment bacteria are effective at mineralizing organic matter, both aerobically and anaerobically [16], [17]. Recent reports illustrated that microbial communities in sediments have wide capacities for degrading high-molecular-weight (HMW) substrates and can hydrolyse a broader range of substrates than those in seawater from the same location [18]–[20]. Interestingly, a recent study showed that phosphorus was released from organic compounds by microbes using phosphatase to relieve limitation of utilizable carbon in the organic-rich and anoxic Baltic Sea sediments [21].

Although bacteria are key players in mineralization interlinked with spatial and environmental factors, the whole bacterial community composition in the sediment [22]–[25] and its role played in nutrient effluxes, such as release of organic phosphorus from sediment, has been less studied [25]. However, our recent study [25] emphasized the associations between bacterial community composition and different chemical forms of phosphorus, rather than focusing organic phosphorus, in brackish Baltic Sea sediments, using multivariate methods.

The bottom sediments of the Gulf of Finland are rich in organic matter, particularly on the eastern side, and consequently hypoxia induces the release of iron-bound phosphate and other effluxes (e.g. ammonium and manganese (Mn)) [8], [9], [14], [26]. Furthermore, Lukkari et al. [9] found the abundant organic phosphorus reserve in hypoxic sediments of the open Gulf and concluded that they can release phosphorus into the water over the long term. Under hypoxic conditions, iron oxyhydroxides are not able to bind phosphate released in mineralization (e.g. in [27]). Thus, bacterial communities degrading sediment organic matter, including organic phosphorus, can be crucial to release of phosphorus in the Gulf of Finland.

We determined the entire bacterial community composition across three gradients in the Gulf of Finland: 1) horizontally from west to east along a continuum of increasing content of carbon in the sediment often associated with high release of phosphorus, 2) from the estuary to the open sea, as well as 3) vertically along the mineralization gradient from the sediment surface to the deeper layers. We assumed that the abundance and utilizability of organic matter, as well as local and regional environmental conditions (e.g. water depth, sedimentation rate, oxygen, and salinity) shape the sediment bacterial community composition, which, in turn, could contribute to the nutrient releases from sediment organic matter to the water column. We used terminal restriction fragment length polymorphism (T-RFLP) and cloning of the bacterial 16S rRNA gene with up-to-date multivariate statistics to examine bacterial community structure along environmental and spatial heterogeneity. In addition, the associations of individual community members with sediment chemical parameters such as different chemical forms of phosphorus, including two organic fractions, were investigated. We found that the sediment bacterial community composition differentiated horizontally, mainly along organic pollution gradients correlating strongly with organic carbon, nitrogen and phosphorus, and hence seemed to reflect the eutrophic conditions of the Gulf of Finland. The predominance of bacteria participating in terminal mineralization processes, such as sulphate-reducing bacteria producing sulphide, in the most hypoxic part of the Gulf, suggest that bacteria process most of the benthic energy as H_2_S [2] and support development of anoxia.

## Materials and Methods

### Research Area and Sediment Sampling

**Figure 1:**
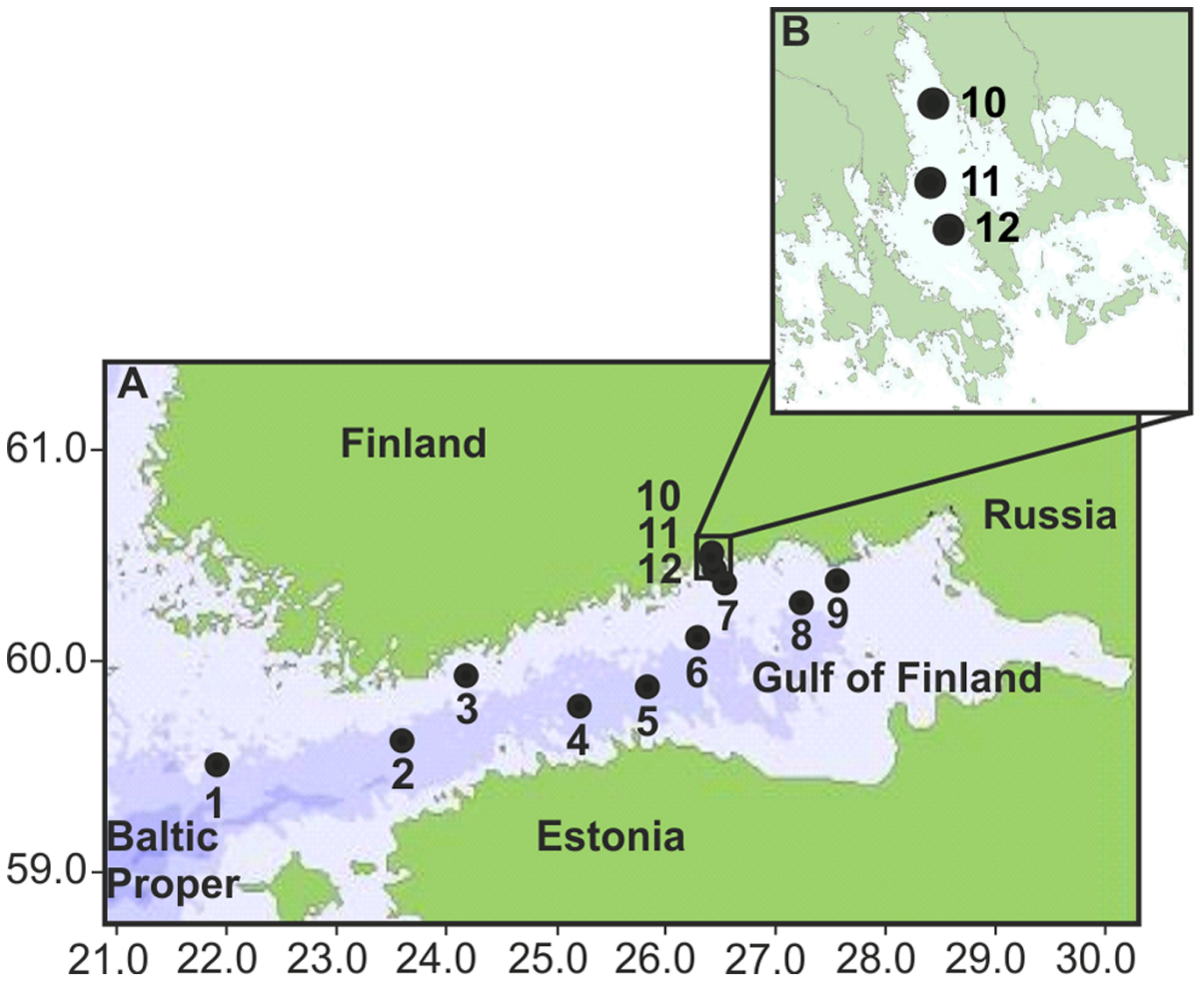
The research area and the sediment sampling sites. (A) Sediment sampling area in the northern Baltic Proper (1), in the Gulf of Finland (2−9), and in Ahvenkoskenlahti Bay (10−12) in the brackish Baltic Sea. The numbers refer to samplings site as follows: 1 = AS7, 2 = JML, 3 = C63, 4 = E3, 5 = GF2F, 6 = LL3A, 7 = Bisa1, 8 = XV1, 9 = BZ1, 10 = AHLA2, 11 = AHLA 6 and 12 = AHLA9. (B) A magnification of Ahvenkoskenlahti Bay and the location of sampling sites in the estuary.

**Figure 2:**
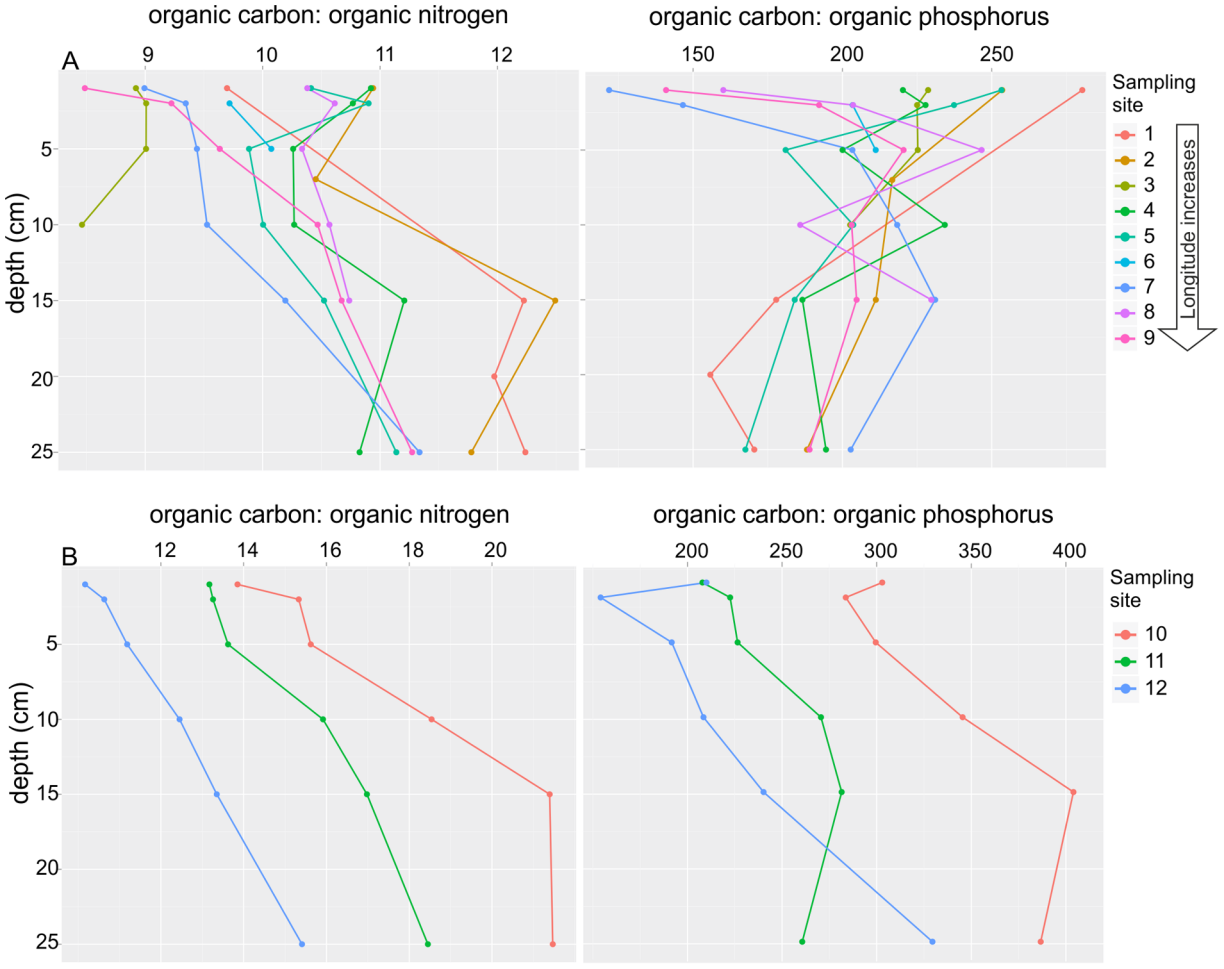
Ratios of organic carbon vs. organic nitrogen and organic carbon vs. organic phosphorus of the sediment samples. (A) Ratios of organic carbon vs. organic nitrogen and organic carbon vs. organic phosphorus along the continuum beginning from the Baltic Proper and western Gulf of Finland towards the eastern Gulf of Finland (sampling sites 1−9), (B) in the Ahvenkoskenlahti Bay estuary (sampling sites 10−12) and along the sediment depth. Refer to locations of the sediment sampling sites in Figures 1A and 1B.

The Gulf of Finland is an organic-rich inner bay, one of the most severely eutrophic and heavily loaded areas of the brackish Baltic Sea [28], which consequently suffers from bottom hypoxia. The hypoxia is affected by the halocline and patchy bottom topography, which hinder the supply of oxygen (O_2_) to the bottom water [29]. The sampling sites were located along the continua from the open Baltic Proper towards the eastern Gulf of Finland (Figure 1A, sites 1−9) and from the inner to the outer Ahvenkoskenlahti Bay estuary (Figure 1B, sites 10–12). Sampling sites 1−9 and 10−12 were numbered in order of increasing concentration of organic carbon, nitrogen and phosphorus in the sediment (Figure 2A and 2B, Dataset S2) [7]–[9]. The high level of organic pollution on the eastern side of the Gulf is associated with extensive nutrient loading from the Neva River [8], [9], [28], [30], [31], and also from the Kymijoki River [32]. In this area, the concentrations of total carbon and nitrogen are practically the same as that of organic carbon and nitrogen [26], [30], and we considered the total concentrations of carbon and nitrogen as organic and use the terms organic carbon and nitrogen. Thus, we assume that the ratios of total carbon to total nitrogen (C:N) and total carbon to total organic phosphorus (C:P) in relation to the Redfield ratio described changes in the nature of the organic matter, either as a consequence of the preferential degradation of phosphorus and nitrogen compounds or terrestrial versus marine origin [15], [33]. The ratios varied from west to east, from the estuary to the open sea, and from the surface to the deeper sediment layers (Figures 2A and 2B). At the time of sampling, the near-bottom water above sites 1, 2, 4 and 5 was hypoxic (O_2_<2.0 ml l^−1^) and above sites 6 and 8 barely oxic. Sites 1, 4, 5, 7and 8 had white bacterial growth (or remains of it) on the sediment surface and often a smell of hydrogen sulphide (H_2_S) [8], [9]. In contrast, the near-bottom water at sites 3, 7, 9 and 12 was oxic ranging from 3.4 to 8.6 ml l^−1^ O_2_. The bottom water salinity ranged slightly (5.7−9.7 psu) (Table S1). The salinity, oxygen concentrations and other important parameters describing the properties of the near-bottom water and sediments are presented in Table S1. Further details can be found in Lukkari et al. [7]–[9].

The sediments were sampled on cruises of the r/v Aranda (assisted by the r/v Muikku in the estuary) in September 2003 (sites 1 and 2), August 2004 (7−9, 10−12) and April 2004 (sites 3−6). The locations of the sites in the northern Baltic Proper (site 1), the western (sites 2−4), central (sites 5 and 6) and eastern (sites 7−9) Gulf of Finland and in the Ahvenkoskenlahti Bay estuary (sites 10−12) are presented in Figures 1A and 1B. Sites 3 and 7−9 were located on the northern coast of the Gulf whereas sites 1 and 2 and 4−6 were located in the open-sea area. Subsamples of sediment cores were taken from depths of 0−1, 1−2, 4−5, 9−10, 14−15, and 24−25 cm from sites 3−12. Exceptionally, the 24−25 cm layer at the site 8 and 14−15-cm and 24−25-cm layers at the 3 were not sampled. The 8−9-cm instead of the 9−10-cm layer at the site 4 was collected. The 0−1-, 6−7-, 14−15-, 19−20- and 24−25-cm layers below the seafloor were sampled at sites 1–3. Sampling was performed as previously summarized [25] and described in detail at sites 10−12 [7], sites 1−2, 4−6 and 8 [9] and sites 3, 7 and 9 [8].

### Terminal Restriction Fragment Length Polymorphism Analysis and Sequencing of the 16S rRNA Gene

Extraction of total DNA and amplification of the 16S RNA gene by the polymerase chain reaction (PCR) for T-RFLP analysis and for cloning were done, as previously described by Sinkko et al. [25], with the exception of the amount of template DNA in PCR, which was 30 ng. Cloning and sequencing as well as T-RFLP analysis of the amplified 16S rRNA genes were also implemented as previously described [25]. Terminal restriction fragments (T-RFs) of sites 1−3 with lengths between 50 and 700 base pairs (bp) have been published previously [25]. However, here, the T-RFs with lengths between 27 and 700 base pairs (bp) were included in the normalization procedure, which resulted in separate matrices of each T-RF dataset (HaeIII, HhaI, MspI and RsaI in Dataset S1) with the relative abundance of binned T-RFs that were used in statistical analysis.

### Assigning of the 16S rRNA Genes and T-RFs

The 16S rRNA gene sequences were assigned to taxa using a naive Bayesian classifier (version 2.5, RDP training set 9) of the Ribosomal Database Project (RDP) [34]. The 16S rRNA gene sequences have been assigned to the accession numbers from HF912872 to HF913245 in EMBL Nucleotide Sequence Database.

The T-RFs were taxonomically assigned by digesting the 16S rRNA gene sequences *in silico* (virtually) and the 16S rRNA gene clones *in vitro* according to Sinkko et al. [25]. Unlike in our previous work [25], only the HaeIII restriction enzyme was used for *in vitro* identification.

### Chemical Data and Spatial as well as other Properties of the Sampling Sites

Various chemical forms of sedimental phosphorus and elements participating in or relating to phosphorus binding (iron, manganese (Mn), aluminium (Al), calcium (Ca), silicon (Si) and magnesium (Mg)) were previously analysed from phosphorus extraction solutions, as well as the total concentrations of the central elements (P, N, C, S, Fe, Mn, Al, and Ca) of the sediment solid phase [7]–[9]. Six chemical binding or solubility forms of phosphorus were distinguished, using the fractionation method by Jensen and Thamdrup [35], slightly modified by Lukkari et al. [36], [37], and summarized in our previous study [25]. Among all chemical data, parameters that were used in the final models of the multivariate statistical analyses are presented in Dataset S2.

Spatial data comprised of geographical coordinates (Table S1) and sampled sediment depths (Dataset S1). Other properties of the sampling sites included water depth and sediment accumulation rate (Table S1).

### Statistical Analyses

The sediment molecular microbiological (T-RFs) and chemical data as well as other properties of the sampling sites were analysed by distance-based nonparametric multivariate methods [38]–[42], which allows the analysis of non-normally distributed data, including high numbers of zeroes, such as our T-RF data. These permutational methods also enable analysis of data in which the number of variables exceeds the number of observations (here the T-RF n = 134 and sediment sample n = 61), since they first calculate the distance-based principal coordinates, which are used in further analysis. The multivariate analyses, including constrained analysis of principal coordinates (CAP) [40], [43], multivariate multiple regression analysis with and without forward selection [39] and variance partitioning [44], [45], were performed as described [25]. However, the selection of chemical parameters for the final CAP model deviated slightly from our previous study, which excluded collinear and spatially dependent chemical parameters from the final CAP [25]. Here, all significant chemical parameters (*P*<0.01), spatially dependent or not, were fitted in preliminary CAP ordination to detect whether the chemical parameters and T-RFs were linearly dependent. Only linearly behaving noncollinear chemical parameters were included in final the CAP model.

Briefly, the CAP analysis defined the associations between the chemical parameters and individual T-RFs, as well as the bacterial communities. Multivariate multiple regression determined the proportions of variation in the bacterial communities explained by the individual chemical parameters used in the final CAP. Variance partitioning calculated the proportions of variation in bacterial communities explained by the set of chemical variables used in the final CAP model, and the sets of spatial and other underlying covariables such as the properties of the sampling sites as well. Further details can be found in Sinkko et al. [25].

In this study, we also analysed the T-RFs by distance-based discriminant analysis [41] to test whether *a priori* groups of bacterial communities separated spatially, i.e. belonged to estuary, coastal or open-sea sediments, and different depth classes (0−2, 4−7, 8−15, and 19−25 cm below the seafloor). The distance-based discriminant analysis of *a priori* groups was performed in the R environment [46], using the function CAPdiscrim of the package BiodiversityR, with 9999 permutations testing the significance [47]. The function betadisper with 9999 permutations by the function permutest in the package Vegan [43] was used to test the multivariate homogeneity of *a priori* group variances (Figure S1). Both functions CAPdiscrim and betadisper used Bray-Curtis distances calculated between the observations.

## Results

### Structure of the Bacterial Community Composition of Brackish Sediments

**Figure 3:**
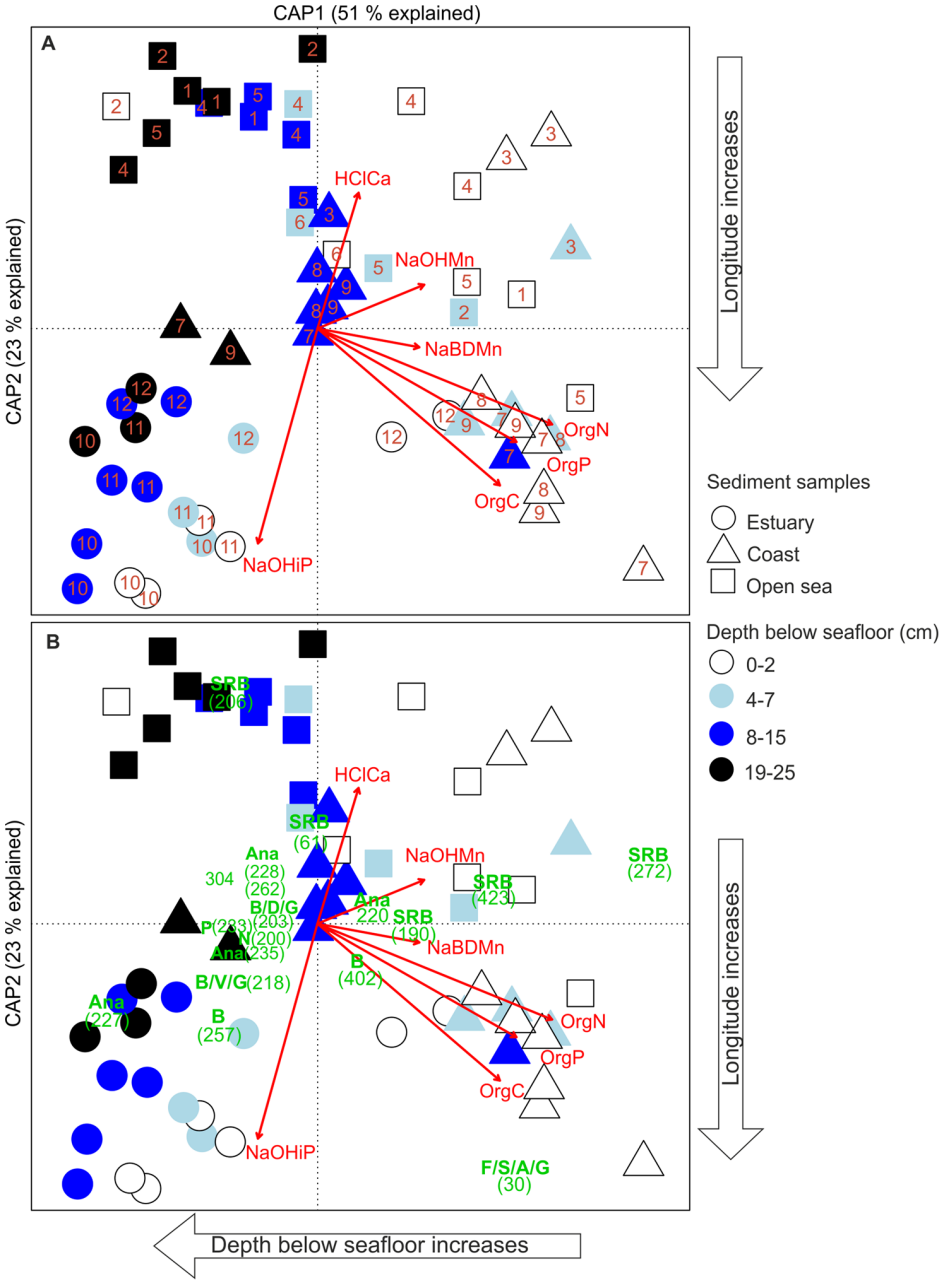
Relationships between bacterial communities and chemical parameters in organic-rich brackish Baltic Sea sediments. (A) Structure of the bacterial community composition constrained by chemical parameters, and (B) associations of bacterial community members with chemical parameters. Constrained analysis of principal coordinates (CAP), using Bray-Curtis distance, was performed on HaeIII terminal restriction fragments (T-RFs, n = 134, refer to Dataset 1 and Table 1 and S2) of the bacterial 16S rRNA genes and chemical parameters (red arrows) of sediment samples (n = 61). The chemical parameters were: HClCa = HCl-extractable calsium, NaBDMn = redox-sensitive (NaBD-extractable) manganese, NaOHMn = NaOH-extractable manganese, NaOHiP = immobile (NaOH-extractable) inorganic phosphorus, OrgC = organic carbon, OrgN = Organic nitrogen, OrgP = organic phosphorus. Numbers (A) on the top of the symbols indicate the sampling sites (refer to Figures 1A and 1B). Numbers in parentheses (B) indicate T-RFs (in bp). The letters below the numbers (B) denote the taxonomic assignments of T-RFs as follows: A = *Alphaproteobacteria*, Ana = *Anaerolineaceae*, B = *Betaproteobacteria*, F = *Flavobacteria*, G = *Gammaproteobacteria*, N = *Nitrospira*, P = *Planctomycetaceae*, S = *Sphingobacteria*, SRB = Sulphate-reducing bacteria, V = *Verrucomicrobia*. Only T-RFs with canonical scores above 0.2 for axes 1 and 2 were included.

Constrained analysis of principal coordinates (CAP) of bacterial 16S rRNA gene T-RFs, produced by HaeIII, and chemical data of sediments in the Gulf of Finland (Figures 1A and 1B) found a strong dependence between bacterial communities and organic nitrogen, carbon, and phosphorus, of which roughly half was labile organic phosphorus. The bacterial community composition of the surface sediments changed horizontally from the western coast (site 3) [8], [9] and open-sea (1−6) sites with less organic pollution towards the organic-rich eastern coast (sites 7−9) [8], [9] and open-sea accumulation basin (site 5) [9] (Figure 3A and Figure S2). In addition, an association of redox-sensitive (NaBD-extractable) Mn and bacterial communities in the coastal and open-sea surface sediments was detected. On the western coast (site 3) and open Gulf (4), some communities were associated with HCl-extractable Ca and NaOH-extractable Mn. The estuary differed from both coast and open sea, since the bacterial communities were associated with phosphorus bound to Al oxides (NaOH-extractable), which decreased from the inner to the outer estuary. The surface sediments at the outermost site (site 12) differed from the other estuary sites and were grouped with the organic-rich eastern coast sites (Figure 3A and Figure S2). In addition to horizontal change, CAP visualized the vertical change in bacterial communities from the surface to the deeper sediment layers (Figure 3A and Figure S2). The bacterial communities from the surface (mainly 0−2 and 4−7 cm), middle (mainly 8−15 cm) and the deepest layers (19−25 cm) were grouped loosely together. The vertical CAP axis roughly separated the surface and the deepest communities, of which the latter were mainly located on the left side of the CAP plot (Figure 3A and Figure S2). In total, CAP analysis explained 42% of the variation in the bacterial communities (*P = *0.0001). CAP analyses, which were based on the T-RFs produced by MspI and RsaI resulted in approximately similar ordinations (Figure S2). Notable part of the T-RFs produced by HhaI was longer than 700 bp and was not included in the CAP analysis, which caused dissimilarities in the ordination (Figure S2).

### Interactions of the Bacterial Community and Chemical Parameters

In the most organic-rich eastern coastal sediments, a T-RF (30 bp) belonging to the classes *Flavobacteria, Sphingobacteria* (phylum *Bacteroidetes*), *Alphaproteo*- and *Gammaproteobacteria* was abundant and correlated positively mainly with the concentrations of organic carbon, phosphorus and nitrogen (Figure 3B). In addition, sulphate-reducing taxa (T-RFs 190, 272 and 423 bp, Table 1) were associated with elevated concentrations of total nitrogen and organic phosphorus (Figure 3B). Along the surface sediments on the coast and in the open sea, sulphate-reducing taxa (T-RFs 190, 271 and 423, Table 1) were mainly associated with elevated concentrations of Mn, mostly with redox-sensitive Mn in the east or central Gulf (site 5), and with the NaOH-extractable Mn on the western side (Figure 3B). In addition, other sulphate-reducing taxa (T-RFS 61 and 206, Table 1) in the western Gulf were associated with acid (HCl)-extractable Ca. In the estuary sediments, bacteria belonging to the phyla *Chloroflexi* (family *Anaerolineaceae*, T-RF 227, Table 1) and *Bacteroidetes* (T-RF 257, Table 1) correlated positively with Al-oxide-bound phosphorus (Figure 3B). In general, T-RFs (e.g. 227, 228, 235, Table 1) of the family *Anaerolineaceae* were prevalent in the deepest layers (15−25 cm).

**Table 1:** HaeIII-digested terminal restriction fragments of 16S rRNA genes, which correlated with the sediment chemical parameters (Figure 3B) of the Baltic Sea and their identification.

		T-RF size (bp)			
	expected^a^	observed^b^		Identification	
clone		1	2	Class or phylum(p)	lowest rank
4–96	38	27^c^	28	*Flavobacteria*	*Flavobacteriaceae* (f)
4–97	38	27^c^	28	*Sphingobacteria*	*Sphingobacteriales* (o)
4–66	38	27^c^	28	*Bacteroidetes* (p)	*Bacteroidetes* (p)
4–61	38	27^c^	28	*Bacteroidetes* (p)^d^	*Bacteroidetes* (p)
10–22	38	27^c^	28	*Alphaproteobacteria*	*Methylocystis* (g)
10–59	38	27^c^	28	*Alphaproteobacteria*	*Alphaproteobacteria* (c)
4–15	38	27^c^	28	*Alphaproteobacteria*	*Pseudorhodobacter* (g)
4–51	38	27^c^	28	*Alphaproteobacteria*	*Rhodobacteraceae* (f)
4–57	38	27^c^	28	*Gammaproteobacteria*	*Gammaproteobacteria* (c)
4–65	38	28^c^	28	*Sphingobacteria*	*Saprospiraceae* (f)
7–135	38	30	30	*Alphaproteobacteria*	*Brevundimonas* (g)
7–154	38	31	31	*Sphingobacteria*	*Haliscomenobacter* (g)
7–60	38	31	31	*Gammaproteobacteria*	*Pseudomonas* (g)
7–26	38	30	30	*Flavobacteria*	*Flavobacterium* (g)
7–137	38	30	30	*Flavobacteria*	*Flavobacteriaceae* (f)
7–4	38	30	30	*Alphaproteobacteria*	*Roseicyclus* (g)
7–181	38	30	30	*Gammaproteobacteria*	*Haliea* (g)
7–147	67	61	60/61	*Deltaproteobacteria*	*Desulfobacterium* (g)
7–73	191	190	190	*Deltaproteobacteria*	*Desulforhopalus* (g)
10–24	192	190	190	*Actinobacteria*	*Acidimicrobineae* (so)
7–13	192	190	190	*Deltaproteobacteria*	*Desulfobacterales* (o)
4–32	203	200	200	*Nitrospira*	*Nitrospira* (g)
4–52	203	202	203	OD1 (candidate phylum)	OD1 (candidate phylum)
10–20	203	203	203	*Bacilli*	*Pasteuria* (g)
4–82	204	202	203	*Deltaproteobacteria*	*Deltaproteobacteria* (c)
7–25	205	203	203	*Gammaproteobacteria*	*Xanthomonadaceae* (f)
7–17	206	204	204	*Deltaproteobacteria*	*Desulfobulbaceae* (f)
4–21	206	205	206	*Deltaproteobacteria*	*Desulfobulbaceae* (f)
4–14	208	206	206	OD1 (candidate phylum)	OD1 (candidate phylum)
10–82	208	206	206	*Deltaproteobacteria*	*Desulfobacteraceae* (f)
7–112	218	217	218	*Gammaproteobacteria*	*Gammaproteobacteria* (c)
7–101	218	218	218	*Betaproteobacteria*	*Methylophilaceae* (f)
10–90	220	218	218	*Betaproteobacteria*	*Oxalobacteraceae* (f)
10–93	220	218	218	*Betaproteobacteria*	*Thiobacillus* (g)
7–119	220	218	218	*Gammaproteobacteria*	*Gammaproteobacteria* (c)
10–60	222	218	218	*Verrucomicrobia* (p)	Subdivision3
10–40	219	219	220	*Anaerolineae*	*Anaerolineaceae* (f)
10–2	227	227	227	*Anaerolineae*	*Anaerolineaceae* (f)
10–69	227	227	227	*Anaerolineae*	*Anaerolineaceae* (f)
4–16	227	228	228	*Anaerolineae*	*Anaerolineaceae* (f)
7–165	228	225	226	*Anaerolineae*	*Anaerolineaceae* (f)
4–38	228	228	228	*Anaerolineae*	*Anaerolineaceae* (f)
7–86	233	232	233	*Planctomycetacia*	*Planctomycetaceae* (f)
10–62	235	235	235	*Anaerolineae*	*Anaerolineaceae* (f)
10–5	240	235	235	*Proteobacteria* (p)	*Proteobacteria* (p)
10–48	257	256	257	*Bacteroidetes* (p)^d^	*Ohtaekwangia* (g)
4–35	257	256	257	*Clostridia*	*Ruminococcaceae* (f)
10–91	259	258	257	*Sphingobacteria*	*Bacteroidetes* (p)
4–63	262	262	262	*Anaerolineae*	*Anaerolineaceae* (f)
4–60	272	271	272	*Deltaproteobacteria*	*Desulfobacula* (g)
7–62	272	272	272	*Deltaproteobacteria*	*Desulfobacula* (g)
7–204	423	423	423	*Deltaproteobacteria*	*Desulfobacula* (g)

T-RF = terminal restriction fragment, bp = base pairs.

a Expected T-RFs based on virtual digestion of partial (appr. 400−500 bp) 16S rRNA gene sequences.

b Observed T-RFs (27−700 bp) produced by terminal restriction fragment length polymorphism analysis of (1) 16S rRNA gene clones and (2) 16S rRNA genes of sediment samples.

c Shift of 10 bp between expected and observed T-RFs was due to conditions in some of the capillary gel electrophoresis runs of digested 16S rRNA gene clones. Therefore, assignments of all T-RFs with observed lengths of 27−31 bp were used to identify T-RF 30 bp in Figure 3B, derived from the sediment samples.

d *Bacteroidetes* incertae sedis.

Multivariate multiple regression analysis specified that among the chemical parameters used in CAP, organic nitrogen explained most of the variation (18%) in bacterial community composition (Table 2, marginal tests). Organic nitrogen and carbon as well as Al-oxide-bound and organic phosphorus together represented 38% of the total variation (Table 2, sequential tests). The proportions of organic carbon, nitrogen, and phosphorus considerably overlapped due to mutual correlations, and as a consequence the proportion of organic phosphorus decreased in sequential tests. Marginal tests, in which each chemical parameter was fitted individually, showed that the proportion of organic phosphorus followed the proportions of organic carbon and nitrogen (Table 2).

**Table 2:** Effects of individual chemical parameters used in CAP analysis on bacteria community composition in the brackish sediment samples.

Marginal tests					
Variable	SS(Trace)	Pseudo-F	P-value	Proportion^1^	
Organic nitrogen	16022.73	12.743	0.0001	0.18	
Organic carbon	12342.11	9.3518	0.0001	0.14	
Organic phosphorus	12777.61	9.7362	0.0001	0.14	
Al-oxide-bound-phosphorus	7174.036	5.0975	0.0003	0.08	
HCl-extractable Ca	3688.42	2.52	0.0164	0.04	
NaOH-extractable Mn	4406.01	3.03	0.0047	0.04	
NaBD-extractable Mn	3814.452	2.605	0.0247	0.04	
**Sequential tests**					
**Variable**	**SS(Trace)**	**Pseudo-F**	**P-value**	**Proportion** 2	**Cumulative proportion** 2
Organic nitrogen	16022.73	12.74	0.0001	0.18	0.18
Organic carbon	9549.55	8.57	0.0001	0.11	0.29
Al oxide-bound-phosphorus	3884.38	3.64	0.0016	0.04	0.33
organic phosphorus	3011.00	2.92	0.0054	0.03	0.36
NaBD- extractable Mn	1652.36	1.62	0.1104	0.02	0.38
HCl-extractable Ca	2890.22	2.93	0.0028	0.03	0.41
NaOH-extractable Mn	2156.75	2.24	0.0161	0.02	0.43

The distance-based multivariate multiple regression analysis was performed on terminal restriction fragments of bacterial 16S rRNA genes produced by HaeIII and sediment chemical parameters.

1 Proportion of each chemical parameter in the variation in bacterial communities.

2 Cumulative proportion of chemical parameter in the variation in bacterial communities.

**Figure 4:**
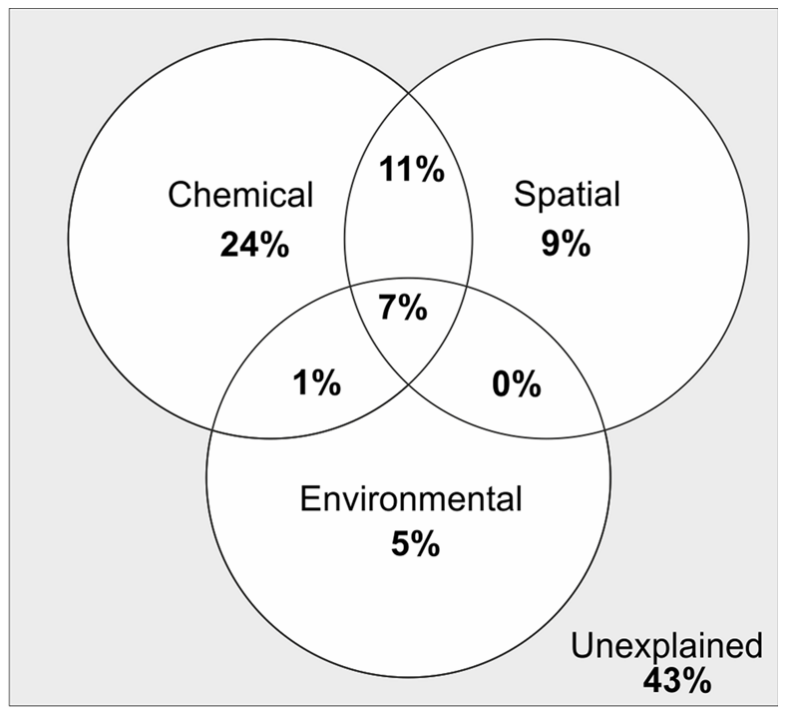
Proportional effects of sediment chemical and spatial factors as well as other properties of the sampling sites on the variation in bacterial communities in organic-rich brackish sediments. Chemical parameters included those used in CAP (refer to Figure 3 and Dataset S2). Spatial parameters included geographic coordinates and sediment depth, and other properties of the sampling sites consisted of sediment accumulation rate (SAR) and water depth (refer to Table S1).

Despite the both horizontally and vertically structured bacterial community composition visualized by CAP, variance partitioning showed that purely chemical parameters explained 24% of the variation in the bacterial communities (Figure 4), whereas purely spatial parameters comprised of geographical coordinates and sediment depth explained 9% of it. The pure proportion of other sampling site properties, consisting of sedimentation rate and water depth, together explained only 5% of the variation. However, the chemical parameters in part were spatially structured and therefore 11% of the bacterial variation was explained by shared chemical and spatial effects (Figure 4). In all, 7% of the bacterial variation was shared by chemical and spatial factors as well as properties of the sampling sites.

### Variation in Bacterial Communities Horizontally in Estuary, Coastal and Open-sea Sediments, and Vertically with Depth

**Figure 5:**
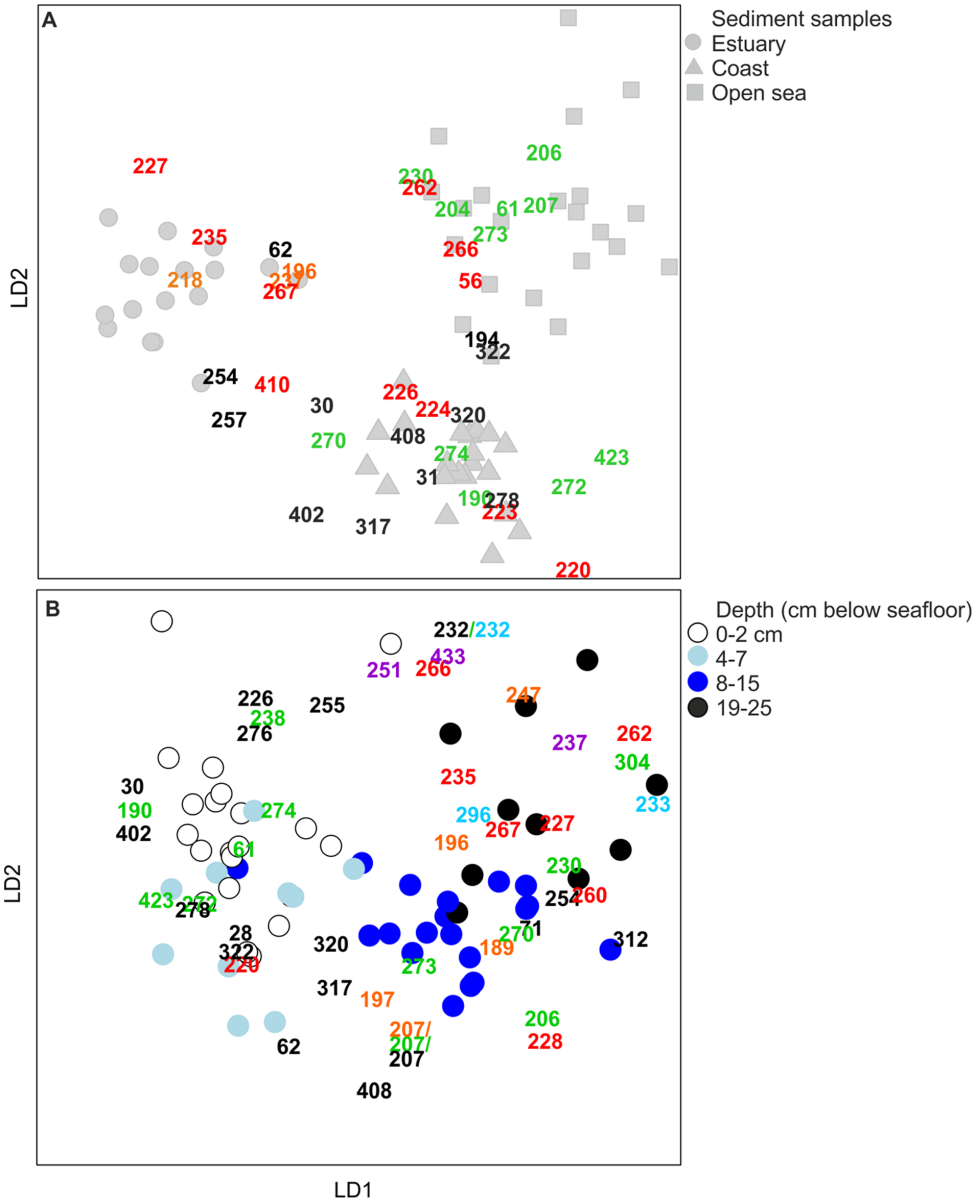
Characteristic T-RFs of bacterial communities in the estuary, coastal and open-sea sediment samples and in the sediment samples from different depths. Distance-based discriminant analysis was performed on bacterial T-RF data (produced by HaeIII), which were divided into *a priori* groups of (A) estuary, coastal and open-sea sediments as well as (B) depth classes. Taxonomic assignments of T-RFs (refer to Table S2): Black numbers = *Alphaproteobacteria*, *Flavobacteria*, *Gammaproteobacteria* and/or *Sphingobacteria* (each T-RF represented more than one taxon), red numbers = *Chloroflexi* (mainly family *Anaerolineaceae*), green numbers = *Deltaproteobacteria*; sulphate reducing taxa, orange numbers = *Betaproteobacteria*, violet numers = *Clostridia*, and light blue numbers = *Planctomycetes*. Only those T-RFs that affected the differentiation of a *priori groups* (canonical scores of discriminant axes 1 and 2 were above 1.0) and which belonged to the most common bacterial groups in each *a priori* group were included.

Since the bacterial 16S rRNA gene T-RFs representing bacterial communities of estuary, coastal and open-sea sediments as well as depth classes (0−2, 4−7, 8−15, and 19−25 cm) were loosely grouped together in CAP, they acted as a *priori* groups in distance-based discriminant analysis. The discriminant analysis, which investigated whether a *priori* groups differed significantly, showed that distinct groups of estuary, coastal and open-sea bacteria were formed with correct classification of 95% (*p*-value of 0.0001)(Figure 5A), and that depth classes also discriminated by correct classification of 64% (*p* = 0.0001) (Figure 5B).

In contrast to CAP analysis, which showed only some of the T-RFs, i.e. those correlating with the chemical parameters selected, the discriminant analysis visualized all T-RFs thriving in the ecosystems studied. It specified that bacterial taxa of classes *Flavobacteria* and *Sphingobacteria* as well as the classes *Alphaproteo*- and *Gammaproteobacteria,* such as the orders *Rhodobacterales, Rhizobiales, Methylococcales, Legionellales* and *Pseudomonadales* predominated on the shallow coast (Figure 5B). Sulphate-reducing taxa (class *Deltaproteobacteria*) and phylum *Cloroflexi,* representing the family *Anaerolineaceae,* were abundant as well (Figure 5A, Table S2). In the open-sea sites, with deeper water and sediments overlain by hypoxic or nearly anoxic near-bottom waters, sulphate-reducing taxa clearly prevailed (Figure 5A). Most of the sulphate reducers were identified as the genera *Desulfobacula* or *Desulfobacterium* (family *Desulfobacteraceae*, Table S2). In the estuary sediments, T-RFs belonging to the family *Anaerolineaceae* and class *Betaproteobacteria* were typical (Figure 5A, Table S2).

Vertically, the classes *Flavo-, Sphingo-*, *Alphaproteo*- and *Gammaproteobacteria* occurred most abundantly in the surface sediments or nearby (0−7-cm depth layers) (Figure 5B, Table S2). The sulphate-reducing taxa, in contrast, were abundant down to 20 cm. In the deepest layers (19−25 cm), the T-RFs of the sulphate reducers began to decrease, and the T-RFs of the family *Anaerolineaceae* were most abundant (Figure 5B, Table S2).

### Identification of Bacterial Taxa

**Figure 6:**
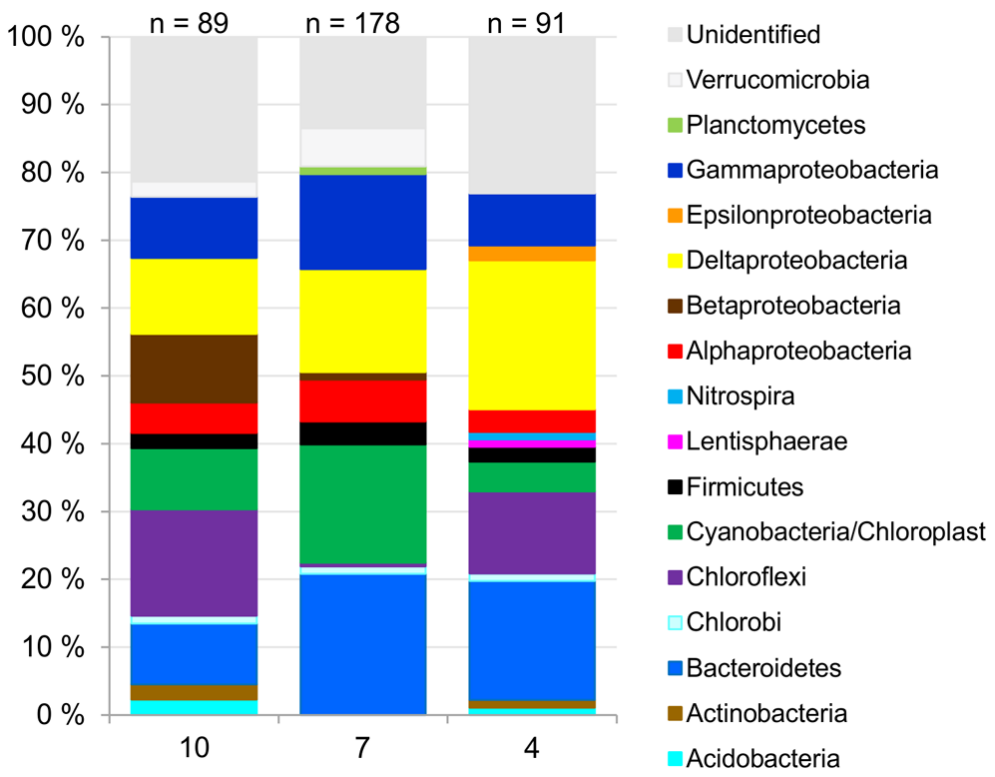
Bacterial taxa of brackish sediments from the organic-rich Gulf of Finland of the northeastern Baltic sea. The 16S rRNA genes were cloned from the surface sediments of the estuary (10), eastern coastal (7) and western open-sea sites (4).

The 16S rRNA genes from the surface sediment (0−1 cm) of the estuary (10), coastal (7) and open-sea areas (4) were cloned to identify the bacterial taxa in the sediments (Figure 6). The cloned and assigned 16S rRNA gene sequences were also used to identify the T-RFs that represented the complete bacterial communities in the sediment samples (Dataset S1, Table S2). The 16S rRNA gene sequence data outlined mainly the same dominant bacterial groups that emerged in the discriminant analysis and CAP. The sequence data also brought out that the *Deltaproteobacteria* sequences, which were common in all libraries, represented only the sulphate reducers in the open-sea library whereas in the estuary other than sulphate-reducing taxa were prevalent.

## Discussion

CAP analysis highlighted that the bacterial communities changed along the gradient of increasing organic nitrogen, carbon and phosphorus from the less polluted towards the most organic-rich sediments, located mainly on the eastern coast of the Gulf of Finland. On the shallow eastern coast, which receives riverine loadings of nutrients and organic matter [8], [9], [30], [31], high production and rapid sedimentation resulted in abundant inputs of fresh organic material into the sediment surface [8]. At the western open-sea sites, however, the mineralization processes at the corresponding sample depths had already proceeded further than those at the eastern sites [9]. This could also be supported by the lower organic C:N and C:P ratios (Figure 2A) on the eastern coast than at the western open-sea sites [8], [9]. Considering the concurrent change in the quantity and quality of organic matter as well as in the sediment bacterial community composition, it is likely that the quality and quantity of organic matter at least partly drove the observed changes in the bacterial community composition.

Among the sediment chemical and spatial factors as well as the properties of the sampling sites, chemical parameters explained most of the variation in the sediment bacterial communities (24%) and, thus, were considered the main driver of the change in bacterial community composition. Of the chemical parameters, organic nitrogen and carbon, Al-oxide-bound phosphorus, probably originating from land and thus indicating presence of erosion-transported clayey material, and total organic phosphorus explained a considerable percentage (36%) of the variation in bacterial community composition. As determined by variance partitioning, chemical parameters were in part spatially structured, which resulted in the spatially structured bacterial communities shown in CAP analysis.

In the distance-based discriminant analysis, the bacterial communities were divided between the estuary, coastal and open-sea sediments, varying environmentally, spatially and, consequently, chemically, as well as between depth classes. The firm grouping, especially between the estuary, coast and open sea, suggested that they are divergent ecosystems, although they are dependent on each other to a certain extent, due to riverine transport, water circulation, production and transport of organic matter and, subsequently, chemical and bacterial composition in the sediment.

### Bacterial Community Composition varies by Sediment Chemistry, Geography and Deposition Environment; Presumptively Initial Organic Matter Degraders were Favoured in Organic-rich Coastal Surface Sediments

The discriminant and CAP analyses using T-RFs showed that classes *Flavo-*, *Sphingo-*, *Alphaproteo*- and *Gammaproteobacteria* predominated in the surface sediments, especially in those of the eastern coast, where these bacteria were strongly associated with elevated concentrations of organic carbon, nitrogen and phosphorus, which indicated an abundance of organic matter. The association suggests that these bacterial groups, including a large number of aerobes or facultative anaerobes (e.g. in [48]–[53]), potentially benefited from the abundant organic matter in the surface sediments overlaid by oxic near-bottom water, and may have intensively decomposed it.

Representatives of the phylum *Bacteroidetes,* such as class *Sphingobacteria* and particularly *Flavobacteria*, are important in degradation of biopolymers in sedimentary organic matter [49], [54]–[56] such as HMW organic compounds [57]. It has also been reported that *Alphaproteobacteria* and *Gammaproteobacteria* have been found in organic-rich sediments, such as those at fish farms, or have been provoked by organic enrichment [58]–[61], and even by complex humic compounds [62]. The abundant decomposing organic matter and oxygenic bacteria detected in our study, and in previous studies, may contribute greatly to nutrient release such as phosphate and ammonium from sediments, either directly from organic matter or indirectly, enhancing hypoxia and hypoxia-induced nutrient release (e.g. in [3]).

As an indication of hypoxia-induced nitrogen recycling [3], dissimilatory nitrate reduction to ammonium (DNRA) predominated in low-oxygen sediments of the Gulf of Finland [63]. Furthermore, either hypoxia-induced or organic matter-derived elevated concentrations of phosphate and ammonium were detected in the hypoxic and even in the oxic near-bottom water of the Gulf of Finland at the time of sampling (sites 1−2, 4−5, 7−9, Table S1) [8], [9].

Below the surface, in the middle depth layers of the outer estuary (4−15 cm), T-RF 257 of the phylum *Bacteroidetes* correlated positively with Al-oxide-bound phosphorus, which is a sign of burial of river-transported material, which in this estuary includes organic matter, probably associated partly with oxide surfaces. If bacterium belonging to the phylum *Bacteroidetes* is considered important in organic matter degradation [49], [54]–[56], [64], the correlation suggests that it can participate in the degradation in river- transported organic matter in bioturbated (personal communication with A. Kotilainen) sub-surface sediment depth layers.

### Sulphate Reducers Predominated in the Organic-rich and Hypoxic Open-sea Sediments

Discriminant analyses and CAP showed that sulphate-reducing taxa (*Deltaproteobacteria*) were abundant in the coastal and the open-sea sediments, even in the surface layers, where the genera *Desulforhopalus* (family *Desulfobulbaceae*, T-RF 190) and particularly *Desulfobacula* (family *Desulfobacteraceae*, T-RFs 272 and 423) correlated positively with organic nitrogen and phosphorus. The associations between the genus *Desulfobacula* and *Desulfobacteraceae* with organic-rich sediments, such as those in the Baltic Sea [25] and organic-rich fish farms [64] have also been previously reported. These associations detected in our study and in previous studies between sulphate reducers and organic-rich sediments may reflect the importance of sulphate reducers in terminal mineralization of organic matter. Previous findings of other investigators consistently estimated that sulphate reduction can account for as much as 50% of organic matter degradation in the coastal zone [65], [66], and demonstrated that the availability of labile organic carbon is essential for the activity of sulphate reducers [67].

In the open Gulf, the near-bottom waters in contact with the sediment surfaces were hypoxic (Table S1). Therefore, the abundance of commonly anaerobic sulphate reducers [68] in the surface sediments may be explained by their participation in the mineralization of abundant organic matter, resulting in hypoxic conditions and the lifting up of the sulphidic zone. Previously, Jorgensen et al. [66] observed that in shallow areas with high rates of organic sedimentation, sulphate reduction increased and the sulphate zone was near the sediment surface.

CAP analysis found an association of redox-sensitive Mn (NaBD-extractable) oxide and alkali-extractable Mn (NaOH-extractable, including also Mn associated with organic matter) with *Desulforhopalus* (family *Desulfobulbaceae*, T-RF 190) and particularly *Desulfobacula* (family *Desulfobacteraceae*, T-RFs 423 and 272) in coastal and open-sea surface sediments. The sulphate reducers thus possibly participated in another process involved in organic matter mineralization near the sediment surface: redox cycling of Mn, which is known to be governed by microbes [69], such as sulphate-reducing bacteria, which reduce Mn oxides [70], [71]. As an indication of Mn reduction, elevated concentrations of dissolved Mn (Mn^2+^) were detected in the pore waters of coastal and open-sea surface sediments (sites 3−9, especially 5 and 6, Figure S3), with the parallel abundance of loosely bound Mn in the sediment (Dataset S2) [8], [9], although part of dissolved Mn can originate in organic matter [72]. Diffusion of Mn^2+^ into the bottom water detected by Lukkari et al. [8], [9], [37] (Table S1) may also affect the release of phosphate from the sediment, since phosphate can be associated with Mn oxides [73]. However, whether the genus *Desulfobacula* or *Desulforhopalus* reduced particulate Mn to soluble Mn^2+^ dissimilatively coupled to organic matter oxidation, or the particulate Mn was indirectly reduced e.g. by sulphide produced by *Desulfobacula* or *Desulforhopalus,* remains to be determined since enzymatic Mn reduction by these genera is poorly known.

Sulphate-reducing bacteria could, via organic matter mineralization, indirectly contribute to phosphate release [25], which was detected from reduced sediments of the Gulf of Finland (Table S1) [8], [9]. Sulphides, produced in hypoxic sediments by sulphate reducers, form insoluble ferrosulphides with ferric iron [74] and, consequently, iron is not capable of forming ferric oxyhydroxides to bind phosphate in oxic zones [75]. Hence, iron capture fuelled by sulphate reducers, abundant down to 20 cm below the seafloor in this study, may perhaps contribute to the long-term release of phosphate under hypoxic conditions from organic phosphorus [76], which seemed to be preserved in the deeper open-sea sediments (Figure 2A), as also reported recently in the Baltic Proper [10]. Supporting our conclusion of the long-term phosphate release, Lukkari et al. [9] determined that labile organic phosphorus composed roughly half of the organic phosphorus pool of the sediments studied here, which indicates the presence of degradation products [9].

In the western Gulf, particularly in the deeper sediments, sulphate reducers (T-RFs 206 and 61) of the family *Desulfobacteraceae* were associated with elevated concentrations of acid-extractable Ca, a sign of detrital apatite or other Ca minerals [36]. Given that sulphate reducers act in the terminal phase of organic matter decay [68], the correlation probably reflected indirectly the stage of organic matter.

In general, sulphate-reducing bacteria are ubiquitous in environments with low redox potential [68] promoted e.g. by organic matter and hypoxia, and the availability of sulphate as well as labile organic carbon is essential for the activity of these organisms [42]. In this study, the quality of the organic matter and hypoxia explained the high amount of sulphate reducers along the sediment cores. However, the abundance of *Desulfobacula* [77], [78], which only use sulphate as an electron acceptor, could also partly be due to the slight salinity increase (Table S1), i.e. increased sulphate concentration, in the open-sea sediments, as we recently reported [25].

### Members of the Phylum Chloroflexi were Typical of the Deepest Sediment Layers

Discriminant analysis found that the family *Anaerolineaceae* (phylum *Chloroflexi*) predominated in the deepest layers (19−25 cm) throughout the study area. In the deep layers of the outer estuary, a T-RF (227 bp) representing *Anaerolineaceae* correlated positively with Al-bound phosphorus, which is a sign of phosphorus buried with erosion material. However, Al in alkalic extraction solution could originate also from humic complexes [36], [79] and thus the correlation with relatively immobile Al-oxide-bound phosphate may have been indicated indirectly the presence of terrestrial organic matter [7], [36] and the terminal mineralization process of it, participated in by the bacteria of *Anaerolineaceae*. Family *Anaerolineaceae* consists mainly of anaerobic fermenters [80]–[82] that have been found mostly in organic-rich environments such as anaerobic methanogenic sludges [80], [82], [83] or sediments [24], [84]. Recently, various anaerolineacean bacteria were detected in deep-core layers covering the last 8000 years of the Baltic Sea [85], which suggests that they may contribute to organic matter mineralization processes in the deeper biosphere. There are studies in which H_2_ -producing *Anaerolineaceae* strains grew more rabidly in the presence of hydrogenotrophic methanogens [80], [82], [83]. In the moderately saline sediments of the Baltic hydrogenotrophic methanogenesis has been frequently detected (e.g. [86]–[89]) and may favour those anaerolineacean species that benefit from hydrogenotrophy. However, factors explaining the variation in T-RFs of *Anaerolineaceae* and presumed syntrophy with methanogens in the Baltic Sea remain to be studied.

### Conclusions

Bacterial community structure shifted along the continuum of less polluted towards organic-rich sediments. The shift detected in bacterial communities along the organic pollution gradient most probably contributed to the organic matter-derived release of phosphate and ammonium, as reported recently [14]. Hypoxia-induced fluxes of phosphate and ammonium into the bottom water can, if mixed in the photic zone, sustain the eutrophication. Sulphate-reducing taxa, especially of the family *Desulfobacteraceae*, predominant in the hypoxic open-sea sediments of the Gulf, participated in phosphate release processes in several different ways. The high number of sulphate-reducing bacteria indicates that the Baltic Sea is in the late phase of progressed hypoxia, characterized by Diaz and Rosenberg [2], where the hypoxic zone has expanded and the upward flow of energy in the food chain is instead directed downwards, feeding sulphate reducers in the sediment. The less hypoxic surface sediment from the coastal area of the Gulf of Finland favoured bacteria that effectively degraded organic matter such as the classes *Flavobacteria*, *Sphingobacteria* and classes *Alphaproteo*- and *Gammaproteobacteria*. Sulphate reducing-taxa, especially of the family *Desulfobacteraceae*, predominated in the hypoxic open-sea sediments of the Gulf. The phylum *Chloroflexi* (family *Anaerolineaceae*) was abundant in the deepest sediments throughout the heavily eutrophic Gulf, suggesting that they may play a role in the deep brackish and organic-rich biosphere, with high production of methane. Baltic Sea sediments could be an ideal place to study microbes participating in methane processes and define the biogeochemical roles of family *Anaerolineaceae* in deep sediments, since anaerobic decomposition via methanogenesis is central [90]. Abundant organic loading in shallow coastal seas not only increases autochthonous production but may also maintain active nutrient recycling by microbes between the sediment and the water column. Additionally, deposits of organic phosphorus may possibly represent long-lasting reserves of eutrophication-inducing phosphate to the system. The effects of organic pollution are thus far-reaching.

## Supporting Information

Figure S1 **Homogeneity of variances in** ***a priori*** **groups used in the distance-based discriminant analysis on bacterial T-RF data.** (A) *A priori* groups of estuary, coastal and open-sea sediments. (B) *A priori* groups of depth classes (refer to Figures 5A and 5B).
(TIF)

Figure S2 **Structure of bacterial community composition constrained by chemical parameters in organic-rich brackish sediments.** Constrained analysis of principal coordinates (CAP), using Bray-Curtis distances, was performed on terminal restriction fragments (T-RFs) produced by (A) HhaI, (B) MspI and (C) RsaI and chemical parameters (red arrows) of sediment samples. The chemical parameters were: HClCa = HCl-extractable calcium, NaBDMn = redox-sensitive (NaBD-extractable) manganese, NaOHMn = NaOH-extractable manganese, NaOHiP = Al-oxide-bound (NaOH-extractable) phosphorus, OrgC = organic carbon, OrgN = organic nitrogen, OrgP = organic phosphorus. Numbers on the top of the symbols indicate the sampling sites (refer to Figures 1A and 1B).
(TIF)

Figure S3 **Concentrations of phosphate (PO_4_-P) and manganese (Mn^2+^) in sediment pore water of the sampled sediments.**
(TIF)

Table S1 Characteristics of the sampled sediments, overlying water column and near-bottom water of the Baltic Sea.
(DOCX)

Table S2 Identification of terminal restriction fragments based on HaeIII-digested 16S rRNA genes of the sediments sampled from the Gulf of Finland.
(DOCX)

Dataset S1 **Abundance of terminal restriction fragments produced by HaeIII, HhaI, MspI, and RsaI in the sampled sediments.**
(XLSX)

Dataset S2 **Concentrations of the chemical parameters used in statistical analyses.**
(DOCX)
