# Supplementary figures and images for: Bacteria Contribute to Sediment Nutrient Release and Reflect Progressed Eutrophication-Driven Hypoxia in an Organic-Rich Continental Sea
Source: PLoS One. 2013 Jun 25;8(6):e67061. doi: 10.1371/journal.pone.0067061 (PMC3692436; doi:10.1371/journal.pone.0067061)

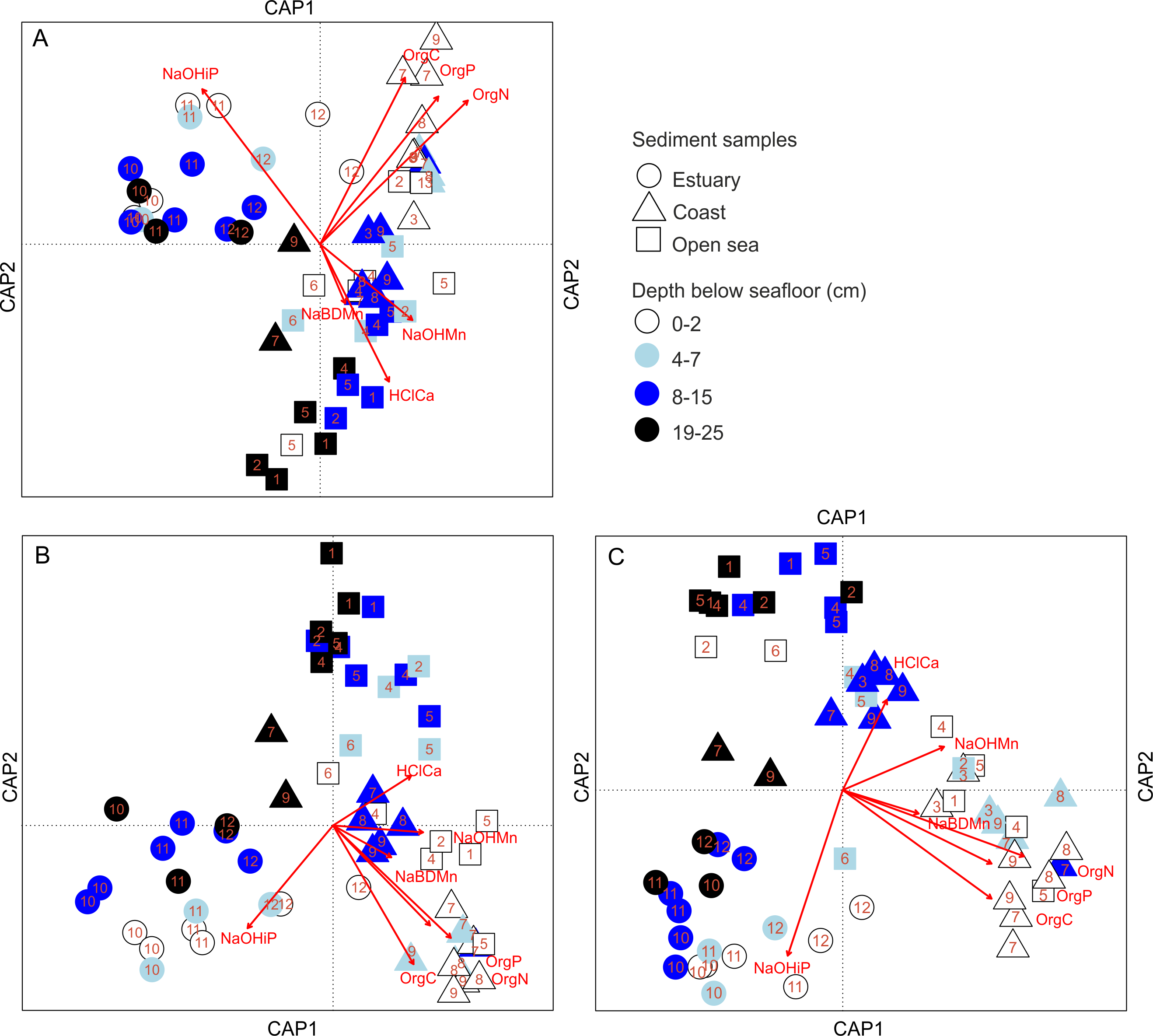

Supplement: Figure S2 — Structure of bacterial community composition constrained by chemical parameters in organic-rich brackish sediments. Constrained analysis of principal coordinates (CAP), using Bray-Curtis distances, was performed on terminal restriction fragments (T-RFs) produced by (A) HhaI, (B) MspI and (C) RsaI and chemical parameters (red arrows) of sediment samples. The chemical parameters were: HClCa = HCl-extractable calcium, NaBDMn = redox-sensitive (NaBD-extractable) manganese, NaOHMn = NaOH-extractable manganese, NaOHiP = Al-oxide-bound (NaOH-extractable) phosphorus, OrgC = organic carbon, OrgN = organic nitrogen, OrgP = organic phosphorus. Numbers on the top of the symbols indicate the sampling sites (refer to Figures 1A and 1B). (TIF) [file pone.0067061.s002.tif]

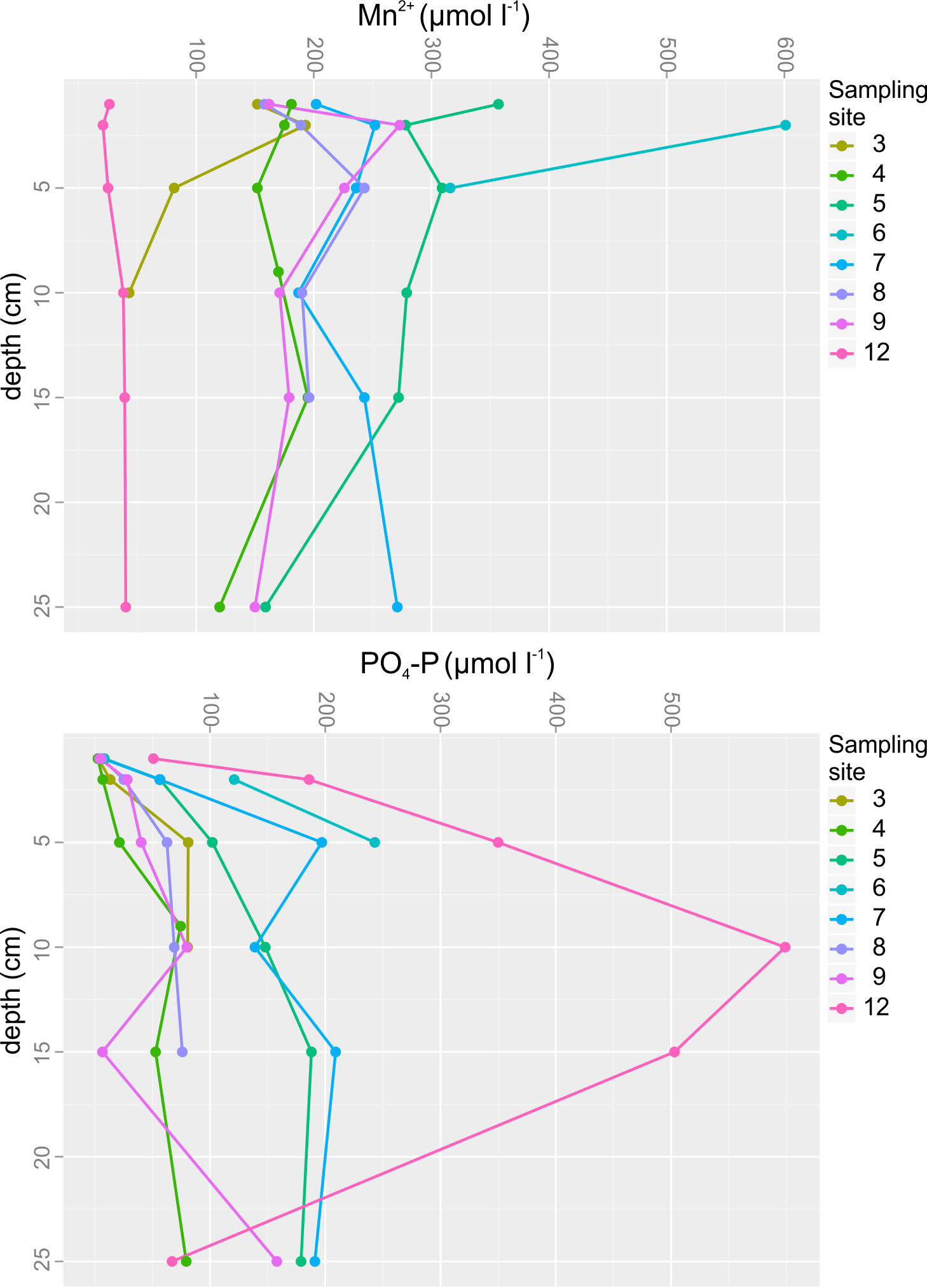

Supplement: Figure S3 — Concentrations of phosphate (PO4-P) and manganese (Mn2+) in sediment pore water of the sampled sediments. (TIF) [file pone.0067061.s003.tif]
