# Supplementary material for: Bacteria Contribute to Sediment Nutrient Release and Reflect Progressed Eutrophication-Driven Hypoxia in an Organic-Rich Continental Sea
Source: PLoS One. 2013 Jun 25;8(6):e67061. doi: 10.1371/journal.pone.0067061 (PMC3692436; doi:10.1371/journal.pone.0067061)
Supplement: Table S1 — Characteristics of the sampled sediments, overlying water column and near-bottom water of the Baltic Sea. (DOCX) [file pone.0067061.s004.docx]

**Table S1** Characteristics of the sampled sediments, overlying water column and near-bottom water of the Baltic Sea. Geographical coordinates of sampling sites, redox potentials of the surface and near-surface sediment layers, water depth, sediment accumulation rate (SAR), concentrations of oxygen (O_2_), incubation-derived phosphate (PO_4_-P) flux, manganese (Mn^2+^), ammonium (NH_4_^+^), nitrate (NO_3_^-^) and phosphate (PO_4_-P) as well as salinity in the near-bottom water (5 cm above sediment). Data from Lukkari et al. [1-3].

| Area | Sampling sites^a^ | Coordinates^b^ | | redox potential  (mV)^c^ | | | Water depth (m) | SAR^e^  (g m^-2^ y^-1^) | O_2_  (ml l^–1^) | PO_4_ -P flux (µmol m^-2^d^-2^) | Mn^2+^  (µmol l^–1^) | NH_4_^+^  (µmol l^–1^) | NO_3_^-^  (µmol l^–1^) | PO_4_ -P  (µmol l^–1^) | Salinity  (PSU) |
| --- | --- | --- | --- | --- | --- | --- | --- | --- | --- | --- | --- | --- | --- | --- | --- |
|  |  | latitude | longitude | 1 cm | 2 cm | 5 cm |  |  |  |  |  |  |  |  |  |
| Open | 1 | 59.2800 | 21.5650 | 10 | na | na | 71 | 840 | 1.6 | 198 | 2.2 | 5.8 | 5.5 | 3.4 | 8.6 ^h^ |
|  | 2 | 59.3491 | 23.3760 | 237 | na | -98^d^ | 79 | 354^f^ | 1.8 | -209 | 4.3 | 2.5 | 3.6 | 3.9 | 8.2 ^h^ |
|  | 4 | 59.4495 | 25.1506 | 381 | 309 | -153 | 89 | na | 0.4 | 207 | 16.6 | 8.3 | 1.6 | 3.8 | 9.7 ^h^ |
|  | 5 | 59.5038 | 25.5185 | 182 | -75 | -126 | 84 | 958 | 0.8 | 357 | 2.4 | 2.5 | 6.9 | 3.3 | 9.5 ^h^ |
|  | 6 | 60.0466 | 26.1972 | na | 201 | -42 | 63 | 329 | 2.4 | -245 | 1.6 | 0.8 | 10.4 | 3.1 | 8.2 ^h^ |
| Coast | 3 | 59.4372 | 24.1270 | 391 | 287 | 115 | 45 | 690^g^ | 8.6^h^ | na | 0.4 | 0.6 | 3.3 | 1.0 | 6.0 ^h^ |
|  | 7 | 60.2012 | 26.3455 | -89 | -160 | -196 | 29 | 990 | 3.8 | 1,065 | 5.2 | 8.0 | 5.4 | 2.8 | 5.7 ^h^ |
|  | 8 | 60.1494 | 27.159 | na | -88 | -117 | 58 | 330 | 2.5 | 1,774 | 13.9 | 5.1 | 11.6 | 2.8 | 7.0 ^h^ |
|  | 9 | 60.2464 | 27.3632 | 288 | -39 | -198 | 40 | 430 | 3.4 | 193 | 15.0 | 10.8 | 11.9 | 4.3 | 6.1 ^h^ |
| Estuary | 10 | 60.2731 | 26.2898 | 278 | 264 | 23 | 4 | 730 | 6.4 ^h^ | na | na | 8.5 | 3.6 | 0.9 | na |
|  | 11 | 60.253 | 26.286 | 119 | 92 | 36 | 7 | 660 | 4.3 ^h^ | na | na | 8.6 | 3.1 | 1.6 | na |
|  | 12 | 60.2412 | 26.2973 | 137 | 91 | 235 | 13 | 1,373 | 4.5 | 671 | na | 10.8 | 3.6 | 2.9 | na |

na = not available

^a^The numbers refer to sampling sites as follows: 1 = AS7, 2 = JML, 3 = C63, 4 = E3, 5 = GF2F, 6 = LL3A, 7 = Bisa1, 8 = XV1, 9 = BZ1, 10 = AHLA2, 11 = AHLA 6, and 12 = AHLA9.

^b^WGS84 coordinate system

^c^Considered only suggestive, due to common problems involved in measuring redox potential with electrodes [5].

^d^Exceptionally 7 cm below the seafloor

^e^Sediment accumulation rates originated from Mattila et al. [4].

^f^Average sediment accumulation rate of the sampling site from 1995 to 2003.

^g^Average sediment accumulation rates of the Gulf of Finland were used. Sediment accumulation rate of the sampling site was not available, due to strong erosion at this site.

**^h^**App. 1 m above sediment

**References**

1. Lukkari K, Leivuori M, Hartikainen H (2008) Vertical distribution and chemical character of sediment phosphorus in two shallow estuaries in the Baltic Sea. Biogeochemistry 90: 171–191.
2. Lukkari K, Leivuori M, Vallius H, Kotilainen A (2009) The chemical character and burial of phosphorus in shallow coastal sediments in the northeastern Baltic Sea. Biogeochemistry 94: 141–162.
3. Lukkari K, Leivuori M, Kotilainen A (2009) Trends in chemical character and burial of sediment phosphorus from open sea to organic rich inner bay in the Baltic Sea. Biogeochemistry 96: 25-48.
4. Mattila J, Kankaanpää H, Ilus E (2006) Estimation of recent sediment accumulation rates in the Baltic Sea using artificial radionuclides ^137^Cs and ^239,240^Pu as time markers. Boreal Env Res 11: 95–107.
5. Drever JI (1997) The Geochemistry of Natural Waters: Surface and Groundwater Environments. New Jersey: Prentice-Hall, Inc.
