# Supplementary material for: Bacteria Contribute to Sediment Nutrient Release and Reflect Progressed Eutrophication-Driven Hypoxia in an Organic-Rich Continental Sea
Source: PLoS One. 2013 Jun 25;8(6):e67061. doi: 10.1371/journal.pone.0067061 (PMC3692436; doi:10.1371/journal.pone.0067061)
Supplement: Table S2 — Identification of terminal restriction fragments based on HaeIII-digested 16S rRNA genes of the sediments sampled from the Gulf of Finland. (DOCX) [file pone.0067061.s005.docx]

**TableS2.** Identification of terminal restriction fragments based on HaeIII-digested 16S rRNA genes of the sediments sampled from the Baltic Sea.

| T-RF size (bp) | | | |  |  | |
| --- | --- | --- | --- | --- | --- | --- |
|  | expected^a^ | observed^b^ | | identification | | |
| clone |  | 1 | 2 | class | lowest rank | |
| 4-96 | 38 | 27^c^ | 28 | *Flavobacteria* | | *Flavobacteriaceae* (f) |
| 4-97 | 38 | 27^c^ | 28 | *Sphingobacteria* | | *Sphingobacteriales* (o) |
| 4-66 | 38 | 27^c^ | 28 | *Bacteroidetes (p)* | | *Bacteroidetes* (p) |
| 4-61 | 38 | 27^c^ | 28 | *Bacteroidetes* incertae sedis | | *Bacteroidetes* (p) |
| 10-22 | 38 | 27^c^ | 28 | *Alphaproteobacteria* | | *Methylocystis* (g) |
| 10-59 | 38 | 27^c^ | 28 | *Alphaproteobacteria* | | *Alphaproteobacteria* (c) |
| 4-15 | 38 | 27^c^ | 28 | *Alphaproteobacteria* | | *Pseudorhodobacter* (g) |
| 4-51 | 38 | 27^c^ | 28 | *Alphaproteobacteria* | | *Rhodobacteraceae* (f) |
| 4-57 | 38 | 27^c^ | 28 | *Gammaproteobacteria* | | *Gammaproteobacteria* (c) |
| 4-65 | 38 | 28^c^ | 28 | *Sphingobacteria* | | *Saprospiraceae* (f) |
| 7-135 | 38 | 30 | 30 | *Alphaproteobacteria* | | *Brevundimonas* (g) |
| 7-154 | 38 | 31 | 31 | *Sphingobacteria* | | *Haliscomenobacter* (g) |
| 7-60 | 38 | 31 | 31 | *Gammaproteobacteria* | | *Pseudomonas* (g) |
| 7-26 | 38 | 30 | 30 | *Flavobacteria* | | *Flavobacterium* (g) |
| 7-137 | 38 | 30 | 30 | *Flavobacteria* | | *Flavobacteriaceae* (f) |
| 7-4 | 38 | 30 | 30 | *Alphaproteobacteria* | | *Roseicyclus* (g) |
| 7-181 | 38 | 30 | 30 | *Gammaproteobacteria* | | *Haliea* (g) |
| 10-56 | 63 | 56 | 55/56/58 | *Anaerolineae* | | *Anaerolineaceae* (f) |
| 7-147 | 67 | 61 | 60/61 | *Deltaproteobacteria* | | *Desulfobacterium* (g) |
| 4-49 | 68 | 62 | 62 | *Epsilonproteobacteria* | | *Sulfurovum*(g) |
| 10-37 | 68 | 62 | 62 | *Gammaproteobacteria* | | *Methylobacter* (g) |
| 4-8 | 68 | 62 | 62 | *Gammaproteobacteria* | | *Gammaproteobacteria* (c) |
| 7-98 | 68 | 63 | 62 | *Betaproteobacteria* | | *Hydrogenophaga* (g) |
| 7-29 | 68 | 63 | 62 | *Gammaproteobacteria* | | *Methylococcaceae* (f) |
| 4-20 | 76 | 70 | 71 | *Gammaproteobacteria* | | *Gammaproteobacteria* (c) |
| 4-90 | 76 | 71 | 71 | *Actinobacteria* | | *Rubrobacteridae* (sc) |
| 4-11 | 137 | 135 | 136 | *Cyanobacteria* | | *GpIIa* |
| 7-116 | 163 | 161 | 162 | *Alphaproteobacteria* | | *Roseomonas* (g) |
| 10-38 | 176 | 172 |  | *Anaerolineae* | | *Anaerolineaceae* (f) |
| 4-4 | 177 | 175 |  | *Acidobacteria_Gp17* | | *Gp17* |
| 7-73 | 191 | 190 | 190 | *Deltaproteobacteria* | | *Desulforhopalus* (g) |
| 10-24 | 192 | 190 | 190 | *Actinobacteria* | | *Acidimicrobineae* (so) |
| 7-13 | 192 | 190 | 190 | *Deltaproteobacteria* | | *Desulfobacterales* (o) |
| 7-83 | 192 | 191 | 192 | *Alphaproteobacteria* | | *Alphaproteobacteria* (g) |
| 7-127 | 194 | 193 | 194 | *Alphaproteobacteria* | | *Rhizobiales* (o) |
| 4-100 | 194 | 193 | 194 | *Gammaproteobacteria* | | *Gammaproteobacteria* (c) |
| 7-184 | 195 | 193 | 194 | *Gammaproteobacteria* | | *Gammaproteobacteria* (c) |
| 7-26 | 195 | 193 | 194 | *Verrucomicrobiae* | | *Luteolibacter* (g) |
| 10-23 | 197 | 191 | 192 | *Betaproteobacteria* | | *Betaproteobacteria* (c) |
| 10-18 | 199 | 189 | 189 | *Betaproteobacteria* | | *Betaproteobacteria* (c) |
| 10-88 | 201 | 195 | 196 | *Betaproteobacteria* | | *Betaproteobacteria* (c) |
| 10-28 | 201 | 196 | 196 | *Betaproteobacteria* | | *Betaproteobacteria* (c) |
| 10-34 | 201 | 197 | 197 | *Betaproteobacteria* | | *Betaproteobacteria* (c) |
| 4-32 | 203 | 200 | 200 | *Nitrospira* | | *Nitrospira* (g) |

T-RF = terminal restriction fragment, bp = base pairs

^a^Expected T-RFs based on virtual digestion of partial (appr. 400−500 bp) 16S rRNA gene sequences.

^b^Observed T-RFs (27−700 bp) produced by terminal restriction fragment length polymorphism analysis of (1) 16S rRNA gene clones and (2) 16S rRNA genes of sediment samples.

^c^Shift of 10 bp between expected and observed T-RFs was due to conditions in some of the capillary gel electrophoresis runs of digested 16S rRNA gene clones. Therefore, assignments of all T-RFs with observed lengths of 27−31 bp were used to identify T-RF 30 bp in Figure 3B, derived from the sediment samples.

**TableS2. (continued)** Identification of terminal restriction fragments based on HaeIII-digested 16S rRNA genes of the sediments sampled from the Baltic Sea.

| T-RF size (bp) | | | |  | |  | | |  |
| --- | --- | --- | --- | --- | --- | --- | --- | --- | --- |
|  | expected^a^ | observed^b^ | | | identification | | | |  |
| clone |  | 1 | 2 | | class | | lowest rank | |  |
| 4-52 | 203 | 202 | 203 | | OD1 (candidate phylum) | | | OD1 (candidate phylum) | |
| 10-20 | 203 | 203 | 203 | | *Bacilli* | | | *Pasteuria* (g) | |
| 4-82 | 204 | 202 | 203 | | *Deltaproteobacteria* | | | *Deltaproteobacteria* (c) | |
| 7-25 | 205 | 203 | 203 | | *Gammaproteobacteria* | | | *Xanthomonadaceae* (f) | |
| 4-21 | 206 | 205 | 206 | | *Deltaproteobacteria* | | | *Desulfobulbaceae* (f) | |
| 4-14 | 208 | 206 | 206 | | OD1 (candidate phylum) | | | OD1 (candidate phylum) | |
| 10-82 | 208 | 206 | 206 | | *Deltaproteobacteria* | | | *Desulfobacteraceae* (f) | |
| 4-33 | 208 | 207 | 207 | | *Deltaproteobacteria* | | | *Desulfonema* (g) | |
| 4-94 | 210 | 208 | 207 | | *Sphingobacteria* | | | *Ferruginibacter* (g) | |
| 10-30 | 211 | 207 | 207 | | *Betaproteobacteria* | | | *Betaproteobacteria* (c) | |
| 7-39 | 215 | 213 | 214 | | *Deltaproteobacteria* | | | *Desulfuromonadaceae* (f) | |
| 4-55 | 217 | 216 | 216 | | *Deltaproteobacteria* | | | *Deltaproteobacteria* (c) | |
| 7-112 | 218 | 217 | 218 | | *Gammaproteobacteria* | | | *Gammaproteobacteria* (c) | |
| 7-101 | 218 | 218 | 218 | | *Betaproteobacteria* | | | *Methylophilaceae* (f) | |
| 10-86 | 219 | 216 | 216 | | *Acidobacteria_Gp16* | | | *Gp16* | |
| 10-40 | 219 | 219 | 220 | | *Anaerolineae* | | | *Anaerolineaceae* (f) | |
| 10-90 | 220 | 218 | 218 | | *Betaproteobacteria* | | | *Oxalobacteraceae* (f) | |
| 10-93 | 220 | 218 | 218 | | *Betaproteobacteria* | | | *Thiobacillus* (g) | |
| 7-119 | 220 | 218 | 218 | | *Gammaproteobacteria* | | | *Gammaproteobacteria* (c) | |
| 10-60 | 222 | 218 | 218 | | *verrucomicrobia* (p) | | | *Subdivision3* | |
| 10-42 | 223 | 221 | 221 | | *Deltaproteobacteria* | | | *Deltaproteobacteria* (c) | |
| 4-3 | 223 | 222 | 223 | | *Anaerolineae* | | | *Anaerolineaceae* (f) | |
| 4-45 | 223 | 223 | 223 | | *Anaerolineae* | | | *Anaerolineaceae* (f) | |
| 10-58 | 224 | 223 | 223 | | *Anaerolineae* | | | *Anaerolineaceae* (f) | |
| 10-55 | 225 | 225 | 224 | | *Anaerolineae* | | | *Anaerolinea* (g) | |
| 10-92 | 226 | 225 | 226 | | *Actinobacteria* | | | *Actinobacteria* (c) | |
| 10-25 | 226 | 226 | 226 | | *Anaerolineae* | | | *Anaerolineaceae* (f) | |
| 7-133 | 226 | 225/226 | 226 | | *Sphingobacteria* | | | *Bacteroidetes* (p) | |
| 10-2 | 227 | 227 | 227 | | *Anaerolineae* | | | *Anaerolineaceae* (f) | |
| 10-69 | 227 | 227 | 227 | | *Anaerolineae* | | | *Anaerolineaceae* (f) | |
| 4-16 | 227 | 228 | 228 | | *Anaerolineae* | | | *Anaerolineaceae* (f) | |
| 7-165 | 228 | 225 | 226 | | *Anaerolineae* | | | *Anaerolineaceae* (f) | |
| 4-38 | 228 | 228 | 228 | | *Anaerolineae* | | | *Anaerolineaceae* (f) | |
| 4-101 | 228 | 228 | 228 | | *Cyanobacteria* | | | GpIV | |
| 10-75 | 230 | 229 | 230 | | *Deltaproteobacteria* | | | *Desulfuromonadales* (o) | |
| 7-128 | 232 | 232 | 232 | | *Alphaproteobacteria* | | | *Acetobacteraceae* (f) | |
| 10-78 | 233 | 231 | 232 | | *Acidobacteria* | | | Gp6 | |
| 7-86 | 233 | 232 | 233 | | *Planctomycetacia* | | | *Planctomycetaceae* (f) | |
| 10-62 | 235 | 235 | 235 | | *Anaerolineae* | | | *Anaerolineaceae* (f) | |
| 7-91 | 237 | 236 | 237 | | *Clostridia* | | | *Clostridiales* (o) | |
| 10-5 | 240 | 235 | 235 | |  | | | *Proteobacteria* (p) | |
| 7-87 | 240 | 238 | 238 | | *Deltaproteobacteria* | | | *Desulfobacterium* (g) | |
| 10-74 | 240 | 239 | 238 | | *Deltaproteobacteria* | | | *Deltaproteobacteria* (c) | |
| 7-70 | 249 | 247 | 247 | | *Verrucomicrobiae* | | | *Verrucomicrobiaceae* (f) | |
| 4-72 | 252 | 246 | 247 | | *Betaproteobacteria* | | | *Proteobacteria* (p) | |
| 4-77 | 253 | 252 | 251 | | *Clostridia* | | | *Ruminococcaceae* (f) | |
| 7-46 | 253 | 254 | 254 | | *Epsilonproteobacteria* | | | *Proteobacteria* (p) | |

T-RF = terminal restriction fragment, bp = base pairs

^a^Expected T-RFs based on virtual digestion of partial (appr. 400−500 bp) 16S rRNA gene sequences.

^b^Observed T-RFs (27−700 bp) produced by terminal restriction fragment length polymorphism analysis of (1) 16S rRNA gene clones and (2) 16S rRNA genes of sediment samples.

^c^Shift of 10 bp between expected and observed T-RFs was due to conditions in some of the capillary gel electrophoresis runs of digested 16S rRNA gene clones. Therefore, assignments of all T-RFs with observed lengths of 27−31 bp were used to identify T-RF 30 bp in Figure 3B, derived from the sediment samples.

**TableS2. (continued)** Identification of terminal restriction fragments based on HaeIII-digested 16S rRNA genes of the sediments sampled from the Baltic Sea.

|  | T-RF size (bp) | | |  |  | | |
| --- | --- | --- | --- | --- | --- | --- | --- |
|  | expected^a^ | observed^b^ | | identification | | | |
| clone |  | 1 | 2 | class | lowest rank | | |
| 10-85 | 254 | 255 | 255 | *Sphingobacteria* | | *Haliscomenobacter* (g) |  |
| 10-48 | 257 | 256 | 257 | *Bacteroidetes* incertae sedis | | *Ohtaekwangia* (g) |  |
| 4-35 | 257 | 256 | 257 | *Clostridia* | | *Ruminococcaceae* (f) |  |
| 10-91 | 259 | 258 | 257 | *Sphingobacteria* | | *Bacteroidetes* (p) |  |
| 10-43 | 261 | 260 | 260 | *Anaerolineae* | | *Anaerolineaceae* (f) |  |
| 4-63 | 262 | 262 | 262 | *Anaerolineae* | | *Anaerolineaceae* (f) |  |
| 7-10 | 265 | 265 | 265 | *Clostridia* | | *Ruminococcaceae* (f) |  |
| 4-26 | 267 | 266 | 266 | *Anaerolineae* | | *Anaerolineaceae* (f) |  |
| 4-68 | 272 | 270 | 270 | *Deltaproteobacteria* | | *Desulfobacula* (g) |  |
| 4-60 | 272 | 271 | 272 | *Deltaproteobacteria* | | *Desulfobacula* (g) |  |
| 7-62 | 272 | 272 | 272 | *Deltaproteobacteria* | | *Desulfobacula* (g) |  |
| 10-21 | 274 | 272 | 273 | *Deltaproteobacteria* | | *Desulfobacteraceae* (f) |  |
| 4-75 | 274 | 273 | 273 | *Deltaproteobacteria* | | *Desulfobacteraceae* (f) |  |
| 7-93 | 274 | 274 | 274 | *Deltaproteobacteria* | | *Desulfobacterium* (g) |  |
| 7-69 | 277 | 276 | 276 | *Flavobacteria* | | *Flavobacteriaceae* (f) |  |
| 4-59 | 279 | 278 | 278 | *Flavobacteria* | | *Flavobacteriaceae* (f) |  |
| 7-100 | 291 | 291 | 291 | *Cyanobacteria* | | GpI |  |
| 4-19 | 295 | 295 | 296 | *Planctomycetacia* | | *Planctomyces* (g) |  |
| 7-123 | 308 | 307 | 308 | *Clostridia* | | *Clostridiales* (o) |  |
| 7-66 | 312 | 310 | 312 | *Gammaproteobacteria* | | *Gammaproteobacteria* (c) |  |
| 7-20 | 312 | 311 | 312 | *Clostridia* | | *Ruminococcaceae* (f) |  |
| 7-82 | 315 | 314 | 314 | *Clostridia* | | *Lachnospiraceae* (f) |  |
| 7-183 | 317 | 317 | 317 | *Sphingobacteria* | | *Haliscomenobacter* (g) |  |
| 7-88 | 318 | 318 | 318 | *Gammaproteobacteria* | | *Gammaproteobacteria* (c) |  |
| 7-15 | 319 | 320 | 320 | *Flavobacteria* | | *Lutibacter* (g) |  |
| 7-11 | 322 | 320 | 320 | *Gammaproteobacteria* | | *Gammaproteobacteria* (c) |  |
| 4-53 | 324 | 322 | 322 | *Gammaproteobacteria* | | *Gammaproteobacteria* (c) |  |
| 4-86 | 393 | 392 |  | *Alphaproteobacteria* | | *Proteobacteria* (p) |  |
| 4-78 | 402 | 401 | 402 | *Flavobacteria* | | *Lutibacter* (g) |  |
| 4-91 | 406 | 405 | 406 | *Flavobacteria* | | *Bacteroidetes* (p) |  |
| 4-54 | 407 | 405 | 406 | *Lentisphaeria* | | *Lentisphaeria* (c) |  |
| 7-95 | 409 | 408 | 408 | *Sphingobacteria* | | *Bacteroidetes* (p) |  |
| 10-39 | 412 | 411 | 410 | *Anaerolineae* | | *Anaerolineaceae* (f) |  |
| 7-204 | 423 | 423 | 423 | *Deltaproteobacteria* | | *Desulfobacula* (g) |  |
| 10-1 | 432 | 430 | 433 | *Clostridia* | | *Acetivibrio* (g) |  |
| 7-35 | 446 | 445 |  | *Verrucomicrobiae* | | *Luteolibacter* (g) |  |

T-RF = terminal restriction fragment, bp = base pairs

^a^Expected T-RFs based on virtual digestion of partial (appr. 400−500 bp) 16S rRNA gene sequences.

^b^Observed T-RFs (27−700 bp) produced by terminal restriction fragment length polymorphism analysis of (1) 16S rRNA gene clones and (2) 16S rRNA genes of sediment samples.

^c^Shift of 10 bp between expected and observed T-RFs was due to conditions in some of the capillary gel electrophoresis runs of digested 16S rRNA gene clones. Therefore, assignments of all T-RFs with observed lengths of 27−31 bp were used to identify T-RF 30 bp in Figure 3B, derived from the sediment samples.
