# Supplementary material for: Bacteria Contribute to Sediment Nutrient Release and Reflect Progressed Eutrophication-Driven Hypoxia in an Organic-Rich Continental Sea
Source: PLoS One. 2013 Jun 25;8(6):e67061. doi: 10.1371/journal.pone.0067061 (PMC3692436; doi:10.1371/journal.pone.0067061)
Supplement: Dataset S2 — Concentrations of the chemical parameters used in statistical analyses. (DOCX) [file pone.0067061.s007.docx]

**Dataset 2** Concentrations of various chemical forms of phosphorus and elements from phosphorus fractionation extracts (phosphorus-binding elements) as well as total element concentrations of sediments that were selected to final CAP model, and used in subsequent multivariate regression and variance partitioning analyses. Data from Lukkari et al. [1-3].

|  |  | Concentrations^a^ | | | | | | | | | |
| --- | --- | --- | --- | --- | --- | --- | --- | --- | --- | --- | --- |
|  |  | Reactive P forms | Immobile P forms | | Elements from P fractionation extracts | | | Total elements | | |  |
|  | solvent: | NaOH^b^ | NaOH^b^ | HCl^c^ | NaBD^d^ | NaOH^b^ | HCl^c^ |  |  |  |  |
| Sampling site | Depth ^(i)^  (cm) | NRP^e^ | iP^f^ | ResP^g^ | Mn | Mn | Ca | OrgP^h^ | Organic C | Organic N |  |
| 1 | 1 | 7.6 | 1.7 | 6.86 | 0.64 | 0.00 | 29.10 | 14.44 | 4047.90 | 417.44 |  |
|  | 15 | 4.9 | 1.4 | 6.87 | 0.87 | 0.08 | 28.97 | 11.81 | 2100.02 | 171.79 |  |
|  | 20 | 4.2 | 1.3 | 6.89 | 0.77 | 0.08 | 29.70 | 11.10 | 1729.00 | 144.41 |  |
|  | 25 | 3.3 | 1.2 | 5.34 | 0.46 | 0.00 | 29.02 | 8.61 | 1467.95 | 119.96 |  |
| 2 | 1 | 11.2 | 1.5 | 13.89 | 1.04 | 0.05 | 48.11 | 25.04 | 6347.81 | 580.21 |  |
|  | 7 | 8.9 | 2.3 | 9.05 | 1.16 | 0.15 | 30.28 | 17.97 | 3893.90 | 372.53 |  |
|  | 15 | 4.9 | 1.6 | 6.89 | 1.66 | 0.09 | 26.93 | 11.84 | 2500.47 | 200.18 |  |
|  | 25 | 4.7 | 0.8 | 6.11 | 1.04 | 0.06 | 31.55 | 10.79 | 2030.03 | 172.37 |  |
| 3 | 1 | 10.7 | 2.0 | 6.04 | 2.71 | 0.03 | 26.50 | 16.71 | 3821.50 | 428.36 |  |
|  | 2 | 11.5 | 2.2 | 5.66 | 0.38 | 0.05 | 23.20 | 17.15 | 3858.96 | 428.36 |  |
|  | 5 | 10.6 | 2.3 | 5.95 | 0.44 | 0.15 | 22.89 | 16.56 | 3729.91 | 414.08 |  |
|  | 10 | 6.7 | 1.4 | 4.94 | 0.13 | 0.16 | 22.86 | 11.63 | 2356.17 | 278.43 |  |
| 4 | 1 | 12.31 | 3.07 | 11.41 | 14.18 | 0.39 | 43.26 | 23.72 | 5224.38 | 478.33 |  |
|  | 2 | 10.39 | 2.36 | 10.52 | 3.05 | 0.16 | 31.72 | 20.92 | 4766.46 | 442.64 |  |
|  | 5 | 8.89 | 2.00 | 8.32 | 3.92 | 0.13 | 40.97 | 17.21 | 3442.68 | 335.55 |  |
|  | 10 | 10.74 | 2.32 | 6.94 | 3.77 | 0.39 | 38.02 | 17.68 | 4142.04 | 403.37 |  |
|  | 15 | 7.25 | 1.80 | 6.47 | 4.89 | 0.33 | 35.93 | 13.72 | 2560.15 | 228.46 |  |
|  | 25 | 3.57 | 2.54 | 6.96 | 4.39 | 0.18 | 42.48 | 10.53 | 2048.12 | 189.19 |  |
| 5 | 1 | 9.73 | 4.18 | 16.98 | 153.49 | 0.79 | 80.13 | 26.71 | 6764.63 | 649.68 |  |
|  | 2 | 9.45 | 2.88 | 11.70 | 55.08 | 0.49 | 67.73 | 21.15 | 5020.40 | 460.48 |  |
|  | 5 | 9.59 | 1.59 | 10.50 | 9.27 | 0.12 | 39.30 | 20.08 | 3634.17 | 367.67 |  |
|  | 10 | 10.95 | 1.74 | 10.27 | 9.72 | 0.16 | 44.23 | 21.22 | 4321.04 | 431.93 |  |
|  | 15 | 10.13 | 2.31 | 7.22 | 2.45 | 0.24 | 37.70 | 17.35 | 3192.91 | 303.42 |  |
|  | 25 | 6.30 | 1.65 | 5.33 | 2.00 | 0.17 | 33.95 | 11.63 | 1948.21 | 174.91 |  |
| 6 | 2 | 14.49 | 3.24 | 12.10 | 8.56 | 0.10 | 28.17 | 26.60 | 5411.71 | 556.86 |  |
|  | 5 | 12.29 | 2.82 | 9.85 | 10.06 | 0.09 | 31.44 | 22.14 | 4674.88 | 464.05 |  |
| 7 | 1 | 53.41 | 5.73 | 21.66 | 14.52 | 0.00 | 13.03 | 75.08 | 9149.95 | 1017.35 |  |
|  | 2 | 32.83 | 4.38 | 17.01 | 7.99 | 0.07 | 21.75 | 49.84 | 7305.80 | 781.75 |  |
|  | 5 | 18.16 | 4.24 | 11.34 | 7.13 | 0.12 | 23.81 | 29.50 | 5998.67 | 635.40 |  |
|  | 10 | 18.96 | 3.79 | 8.61 | 7.58 | 0.31 | 23.03 | 27.57 | 6019.48 | 631.83 |  |
|  | 15 | 13.19 | 3.78 | 8.08 | 9.69 | 0.24 | 25.75 | 21.26 | 4912.16 | 481.90 |  |
|  | 25 | 8.78 | 2.65 | 4.79 | 6.46 | 0.21 | 23.08 | 13.57 | 2751.64 | 242.74 |  |
| 8 | 1 | 33.47 | 4.34 | 29.71 | 5.46 | 0.26 | 21.83 | 63.18 | 10115.73 | 974.51 |  |
|  | 2 | 30.78 | 3.93 | 20.59 | 8.63 | 0.18 | 27.58 | 51.37 | 10457.08 | 985.22 |  |
|  | 5 | 18.78 | 6.38 | 11.74 | 7.27 | 0.19 | 27.48 | 30.52 | 7526.43 | 728.21 |  |
|  | 10 | 18.57 | 3.27 | 7.41 | 0.33 | 0.10 | 18.65 | 25.98 | 4828.91 | 456.91 |  |
|  | 15 | 14.45 | 3.11 | 8.07 | 4.43 | 0.17 | 26.18 | 22.52 | 5174.42 | 481.90 |  |
| 9 | 1 | 47.03 | 6.23 | 18.10 | 11.98 | 0.39 | 23.39 | 65.13 | 9179.09 | 1081.60 |  |
|  | 2 | 24.72 | 3.82 | 12.97 | 8.62 | 0.18 | 22.88 | 37.69 | 7243.36 | 785.32 |  |
|  | 5 | 22.78 | 3.88 | 12.32 | 9.08 | 0.52 | 23.70 | 35.10 | 7738.74 | 803.17 |  |
|  | 10 | 12.23 | 3.17 | 11.32 | 7.24 | 0.13 | 27.67 | 23.55 | 4783.12 | 456.91 |  |
|  | 15 | 12.84 | 3.70 | 10.42 | 6.54 | 0.24 | 27.63 | 23.26 | 4762.30 | 446.21 |  |
|  | 25 | 10.75 | 3.41 | 9.69 | 6.15 | 0.21 | 24.03 | 20.44 | 3863.13 | 342.69 |  |

^a^ µmol g^-1^ dry weight (DW)

^b^ Sodium hydroxide

^c^ Hydrochloric acid

^d^ Sodium dithionite in sodium bicarbonate

^e^ Labile organic phosphorus

^f^ Inorganic phosphorus

^g^ recalcitrant organic phosphorus (ResP)

^h^Total organic phosphorus, which constitute both labile organic (NRP) and recalcitrant organic phosphorus (ResP)

^i^ Depth from sediment-water interface.

|  |  | Concentrations^a^ | | | | | | | | | |
| --- | --- | --- | --- | --- | --- | --- | --- | --- | --- | --- | --- |
|  |  | Reactive P forms | Immobile P forms | | Elements from P fractionation extracts | | | Total elements | | |  |
|  | solvent: | NaOH^b^ | NaOH^b^ | HCl^c^ | NaBD^d^ | NaOH^b^ | HCl^c^ |  |  |  |  |
| Sampling site | Depth ^(i)^  (cm) | NRP^e^ | iP^f^ | ResP^g^ | Mn | Mn | Ca | OrgP^h^ | Organic C | Organic N |  |
| 10 | 1 | 7.20 | 5.60 | 7.49 | 1.45 | 0.00 | 22.33 | 14.69 | 4450.09 | 321.27 |  |
|  | 2 | 7.53 | 5.61 | 8.28 | 0.84 | 0.00 | 22.36 | 15.82 | 4487.55 | 292.71 |  |
|  | 5 | 7.22 | 5.34 | 7.86 | 0.44 | 0.00 | 21.71 | 15.08 | 4516.69 | 289.14 |  |
|  | 10 | 6.03 | 6.32 | 7.19 | 0.61 | 0.00 | 24.42 | 13.22 | 4566.65 | 246.31 |  |
|  | 15 | 5.27 | 7.66 | 6.26 | 0.54 | 0.00 | 22.18 | 11.53 | 4658.23 | 217.75 |  |
|  | 25 | 3.88 | 3.50 | 5.83 | 0.51 | 0.00 | 20.40 | 9.71 | 3754.89 | 174.91 |  |
| 11 | 1 | 10.37 | 8.85 | 9.53 | 1.23 | 0.00 | 24.92 | 19.90 | 4137.87 | 314.13 |  |
|  | 2 | 9.99 | 8.39 | 8.94 | 1.01 | 0.00 | 20.13 | 18.93 | 4212.80 | 317.70 |  |
|  | 5 | 10.93 | 8.71 | 7.31 | 1.02 | 0.00 | 20.89 | 18.25 | 4133.71 | 303.42 |  |
|  | 10 | 8.00 | 7.30 | 7.54 | 1.42 | 0.07 | 19.28 | 15.54 | 4204.48 | 264.15 |  |
|  | 15 | 6.81 | 7.53 | 7.83 | 2.27 | 0.08 | 21.03 | 14.64 | 4121.22 | 242.74 |  |
|  | 25 | 6.36 | 5.84 | 7.80 | 2.94 | 0.08 | 18.81 | 14.15 | 3688.29 | 199.90 |  |
| 12 | 1 | 10.74 | 7.30 | 10.35 | 3.31 | 0.00 | 11.63 | 21.09 | 4429.27 | 435.50 |  |
|  | 2 | 16.80 | 13.65 | 12.51 | 5.40 | 0.00 | 20.11 | 29.31 | 4516.69 | 424.79 |  |
|  | 5 | 11.90 | 14.40 | 10.60 | 5.76 | 0.00 | 21.08 | 22.50 | 4312.71 | 385.52 |  |
|  | 10 | 10.40 | 10.18 | 8.79 | 3.37 | 0.00 | 20.40 | 19.19 | 4000.50 | 321.27 |  |
|  | 15 | 9.82 | 8.82 | 7.44 | 3.13 | 0.35 | 18.07 | 17.26 | 4146.20 | 310.56 |  |
|  | 25 | 9.87 | 10.77 | 2.65 | 4.34 | 0.26 | 17.83 | 12.52 | 4125.39 | 267.72 |  |

^a^ µmol g^-1^ dry weight (DW)

^b^ Sodium hydroxide

^c^ Hydrochloric acid

^d^ Sodium dithionite in sodium bicarbonate

^e^ Labile organic phosphorus

^f^ Inorganic phosphorus

^g^ recalcitrant organic phosphorus (ResP)

^h^Total organic phosphorus, which constitute both labile organic (NRP) and recalcitrant organic phosphorus (ResP)

^i^ Depth from sediment-water interface.

**References:**

1. Lukkari K, Leivuori M, Hartikainen H (2008) Vertical distribution and chemical character of sediment phosphorus in two shallow estuaries in the Baltic Sea. Biogeochemistry 90: 171–191.
2. Lukkari K, Leivuori M, Vallius H, Kotilainen A (2009a) The chemical character and burial of phosphorus in shallow coastal sediments in the northeastern Baltic Sea. Biogeochemistry 94: 141–162.
3. Lukkari K, Leivuori M, Kotilainen A (2009b) Trends in chemical character and burial of sediment phosphorus from open sea to organic rich inner bay in the Baltic Sea. Biogeochemistry 96: 25-48.
